# Engineered sTRAIL-armed MSCs overcome STING deficiency to enhance the therapeutic efficacy of radiotherapy for immune checkpoint blockade

**DOI:** 10.1038/s41419-022-05069-0

**Published:** 2022-07-14

**Authors:** Kevin Chih-Yang Huang, Shu-Fen Chiang, Hsin-Yu Chang, William Tzu-Liang Chen, Pei-Chen Yang, Tsung-Wei Chen, Ji-An Liang, An‑Cheng Shiau, Tao-Wei Ke, K. S. Clifford Chao

**Affiliations:** 1 Department of Biomedical Imaging and Radiological Science, China Medical University, Taichung, 40402 Taiwan; 2 Translation Research Core, China Medical University Hospital, China Medical University, Taichung, 40402 Taiwan; 3 Lab of Precision Medicine, Feng-Yuan Hospital, Ministry of Health and Welfare, Taichung, 42055 Taiwan; 4 Proton Therapy and Science Center, China Medical University Hospital, China Medical University, Taichung, 40402 Taiwan; 5 Department of Colorectal Surgery, China Medical University HsinChu Hospital, China Medical University, HsinChu, 302 Taiwan Taiwan; 6 Department of Colorectal Surgery, China Medical University Hospital, China Medical University, Taichung, 40402 Taiwan; 7 Department of Surgery, School of Medicine, China Medical University, Taichung, 40402 Taiwan; 8 Graduate Institute of Biomedical Science, China Medical University, Taichung, 40402 Taiwan; 9 Department of Pathology, Asia University Hospital, Asia University, Taichung, 41354 Taiwan; 10 Department of Radiation Oncology, China Medical University Hospital, China Medical University, Taichung, Taiwan; 11 Department of Radiotherapy, School of Medicine, China Medical University, Taichung, 40402 Taiwan; 12 School of Chinese Medicine & Graduate Institute of Chinese Medicine, China Medical University, Taichung, 40402 Taiwan

**Keywords:** Radiotherapy, Cancer immunotherapy, Mesenchymal stem cells

## Abstract

Radiotherapy (RT) mainly elicits antitumor immunity via the cGAS/STING axis for type I interferon (IFN) production. However, dysregulation of cGAS/STING constrains radiotherapy-induced antitumor immunity and type I IFN-dependent cell death and is associated with shorter survival of patients with colorectal cancer (CRC). Due to their tumor tropism, mesenchymal stem cells (MSCs) have shown the potential to deliver therapeutic genes for cancer therapy. Here, we showed that MSCs enhance the sensitivity to RT by inducing TRAIL-dependent cell death and remodel the tumor microenvironment by recruiting CD8^+^ immune cells to upregulate PD-L1 in the tumor. By engineering MSCs to express CRC-specific soluble TRAIL via adenovirus-associated virus 2 (AAV2), we found that the therapeutic activity of MSC-sTRAIL was superior to that of MSCs alone when combined with RT. Combined treatment with MSC-sTRAIL and RT significantly reduced cell viability and increased apoptosis by inducing TRAIL-dependent cell death in STING-deficient colorectal cancer cells. MSC-sTRAIL directly triggered TRAIL-dependent cell death to overcome the deficiency of the cGAS/STING axis. Moreover, these combination treatments of MSC-sTRAIL and RT significantly remodeled the tumor microenvironment, which was more suitable for anti-PD-L1 immunotherapy. Taken together, this therapeutic strategy represents a novel targeted treatment option for patients with colorectal cancer, especially cGAS/STING-deficient patients.

## Introduction

Colorectal cancer (CRC) is the third most common cause of cancer worldwide and the second leading cause of cancer-related death in adults [1]. Radiotherapy (RT) is a critical therapeutic component of comprehensive cancer treatment [2]. Approximately half of the patients with cancer would benefit from radiotherapy for local control [3]. However, the RT dose that can be administered safely is limited [4], indicating that combination of alternative therapeutic strategies for cancer treatment are necessary.

Recently, RT-induced DNA damage leads to the formation of micronuclei and double-stranded DNA (dsDNA) fragments that are recognized by cGAS following a loss of nuclear compartmentalization, triggering STING-mediated production of type I IFN and other proinflammatory cytokines for anticancer immunity [5–8]. Innate immune sensing by dendritic cells following radiotherapy is also dominated by the cGAS/STING-dependent pathway, which drives the adaptive antitumor immune response [8–11]. Type I IFNs then interact with the IFNα/β receptor (IFNAR), leading to IFNAR phosphorylation and activation of the JAK/STAT pathway. Activated STAT heterodimers translocate to the nucleus and bind to interferon-stimulated response elements of the promoters of IFN-stimulated genes for antitumor immune responses, including TRAIL [12–15]. However, defects in cGAG/STING induced by epigenetic control evades immune surveillance and attenuate the antitumor immune response in several malignancies, such as colorectal cancer and lung cancer [16–18]. Therefore, the STING pathway suppresses tumorigenesis and elicits antitumor immunity, implying that the inhibition of STING function may be selectively suppressed during cancer development.

Mesenchymal stem cells (MSCs) are a heterogeneous group of progenitor cells with tumor tropism properties that are continuously recruited and integrated into the tumor microenvironment (TME) in response to chemokines such as CXCL12. Within the tumor microenvironment, MSCs exert both proapoptotic and prosurvival effects on tumors and modulate immune functions by altering the secreted cytokine profile [19]. MSCs secrete various cytokines, such as TRAIL and type I IFN, and exert antitumor effects [20–22]. Due to their tumor tropism property, MSCs are extremely attractive for directed cancer therapy. Recent studies have suggested that MSCs exert an inhibitory effect on HCC, breast cancer, pancreatic cancer, and CRC, suggesting that MSCs have potential as novel therapeutic agents, especially when combined with radiotherapy [20, 23–27].

Here, we found that radiotherapy elicited TRAIL-dependent cell death by regulating cGAS/STING signaling. cGAS/STING-mediated type I IFN production triggered TRAIL-dependent cell death. However, TRAIL-dependent cell death was attenuated in cGAS-deficient colorectal cancer cells. The administration of MSCs directly triggered TRAIL-dependent cell death, especially in cGAS-deficient colorectal cancer cells, and increased the therapeutic efficacy of radiotherapy. Therefore, we developed soluble TRAIL-armed MSCs by transducing cells with an adenovirus-associated virus (AAV) expressing soluble TRAIL the colorectal cancer-specific CEA promoter CEA. These armed MSCs directly delivered TRAIL and increased the response of a cGAS-deficient CRC animal model to radiotherapy. Moreover, sTRAIL-armed MSCs also remodeled the tumor microenvironment and triggered PD-L1 upregulation in the tumor to enhance the therapeutic efficacy of anti-PD-L1 immunotherapy. Taken together, these results suggest a therapeutic approach in which sTRAIL-armed MSCs increase the benefits of radiotherapy and immunotherapy by overcoming defective cGAS in patients with CRC.

## Materials and methods

### Cell lines

Human CRC cell lines (HT29, SW480, and HCT116) and the mouse CRC cell line CT26 were obtained from American Type Culture Collection (Manassas, VA, USA). All human cell lines have been authenticated using STR profiling. CRC cells were cultured in RPMI 1640 medium supplemented with 10% fetal bovine serum (FBS) and penicillin–streptomycin (100 U/ml penicillin and 100 mg/ml streptomycin). Cells were maintained at 37 °C in a 5% CO_2_ incubator (Thermo Fisher Scientific, Darmstadt, Germany) using standard protocols. The medium was replaced routinely every 2–3 days. Upon reaching 70–80% confluence, the cells were subcultured. Cells were washed and incubated with serum-free medium for 2 h when subconfluent to prepare the conditioned medium (CM). The medium was discarded, and the cells were incubated with the serum-free medium again. After 48 h, the CM was harvested and centrifuged to remove debris, filtered through a 0.22 μm filter, and stored at −20 °C until use.

Human Wharton’s jelly-derived mesenchymal stem cells were purchased from Bioresource Collection and Research Center (BCRC, No. RM60596, Hsinchu, Taiwan). MSCs were cultured in RPMI 1640 medium supplemented with 10% fetal bovine serum (FBS) and penicillin–streptomycin (100 U/ml penicillin and 100 mg/ml streptomycin). WJMSCs were characterized using short tandem repeat (STR) profiling and flow cytometry by BCRC before we obtained, including CD45, CD34, CD90, CD73, CD105, CD14, CD19, and HLA-DR.

### Colorectal cancer-specific AAV2 vector generation and recombinant AAV2 virus purification

The human soluble TRAIL (aa 114–281) and IFNβ1 (aa 22–187) sequence was generated by PCR and subcloned into a pAAV2-CEA vector, which contained the CEA promoter. The viruses were all produced using the triple transfection method, AAV2-sTRAIL and the helper plasmids pRC2-miR342 and pHelper, in 293 T cells. Seventy-two hours after transfection, the cells were collected by centrifugation, and recombinant AAV2 vectors were produced and purified using an AAVpro® purification kit (6232, Takara, Japan). AAV2 titration was performed using quantitative polymerase chain reaction (qPCR) of vector genomes.

### Western blot analysis

Total lysates (30 μg) were resolved on an SDS–PAGE gel and transferred onto PVDF membranes (Millipore, MA, USA) [28, 29] for immunoblot analyses with the indicated antibodies overnight at 4 °C. Membranes were then probed with HRP-conjugated secondary antibodies for 2 h at room temperature. All antibodies were diluted in T*-*Pro Protein Free Blocking Buffer (BioLion Tech., Taipei, Taiwan). The membrane was then incubated with Immobilon Western Chemiluminescent HRP Substrate (Millipore, CA, USA), visualized using an ImageQuant™ LAS 4000 biomolecular imager (GE Healthcare, Amersham, UK), processed using Adobe Photoshop, and quantified using ImageJ software (NIH, MD, USA). Each blot was stripped with immunoblotting stripping buffer (BioLion Tech.) before incubation with the other antibodies.

The following antibodies were used: cGAS (ab224144, Abcam), STING (#13647, Cell Signaling Technology), cleaved caspase-3 (#9661, Cell Signaling Technology and IR96–401, iReal Biotech.), PARP (#9542, Cell Signaling Technology and IR101–420, iReal Biotech.), caspase-8 (#4790, Cell Signaling Technology), DR4 (A6267, ABclonal), DR5 (A1236, ABclonal) and PD-L1 (ab205921, Abcam and #13684, Cell Signaling Technology).

### qRT-PCR

Total RNA was extracted from cell lines with TRIzol (Invitrogen, CA, USA), quantitated by measuring the absorbance at 260 nm, and then reverse-transcribed into cDNAs using iScript™ Reverse Transcription Supermix (Bio-Rad, CA, USA) according to the manufacturer’s instructions. Primers were designed using the Primer design tool (NCBI, USA) according to sequence information from the NCBI database. qRT-PCR was performed in a final reaction volume of 20 μL with iQ™ SYBR® Green Supermix (Bio-Rad, CA, USA) using the CFX96 Touch Real-Time PCR Detection System (Bio-Rad). All reactions were performed in triplicate for each sample, and GAPDH was employed as a reference gene for normalization. The relative gene expression levels were calculated using the 2^−ΔΔCt^ method. Gene expression levels were compared using the *t-*test.

### Assessments of cell growth and apoptosis

Cell growth was assessed using a CCK-8 assay. Apoptosis and necrosis were assessed using an apoptosis/necrosis detection kit (Enzo Life Sciences, Plymouth Meeting, USA). According to the instructions provided with the kit, the apoptotic and necrotic cells were labeled fluorescently with annexin V-EnzoGold and PI, respectively. The caspase-3 activity was evaluated Caspase-3 Colorimetric Assay Kit (K106, Biovision, CA, USA). The experiments were performed in triplicate.

Terminal deoxynucleotidyl transferase (TdT)-mediated dUTP-biotin nick end labeling (TUNEL) was also performed according to the manufacturer’s protocol (In Situ Cell Death Detection Kit, Fluorescein or TMR Red, Roche, Mannheim, Germany). Tissues mounted on slides were fixed with a 4% paraformaldehyde solution for 30 min at room temperature. Following a rinse with phosphate-buffered saline (PBS), the samples were incubated first with phalloidin-rhodamine for 1 h and subsequently with the TUNEL reaction mixture containing terminal deoxynucleotidyl transferase and fluorescein isothiocyanate-dUTP. Three-μm-thick paraffin sections of heart tissues were deparaffinized by immersion in xylene, rehydrated, and incubated in PBS with 2% H_2_O_2_ to inactivate endogenous peroxidases. Next, the sections were incubated with proteinase K (20 μg/ml), washed with PBS, and incubated with terminal deoxynucleotidyl transferase for 90 min and with fluorescein isothiocyanate-dUTP for 30 min at 37 °C using an apoptosis detection kit (Roche, Mannheim, Germany). Then, the sections were stained with 4,6-diamidino-2-phenylindole to detect cell nuclei via UV light microscopic observations (blue). The samples were analyzed in a drop of PBS under a fluorescence and UV light microscope in this state using an excitation wavelength ranging from 450–500 nm, with detection at wavelengths ranging from 515–565 nm (green). The number of TUNEL-positive cardiac myocytes was determined by counting 3 × 10^5^ cardiac myocytes. All morphometric measurements were performed by at least two individuals independently in a blinded manner.

### Combined MSC and radiotherapy in an animal model

BALB/c mice (female, 4 weeks old) were maintained according to the institutional guidelines approved by the China Medical University Institutional Animal Care and Use Committee [Protocol No. CMUIACUC-2018–167]. Before tumor cell inoculation, mice were randomized into different groups (five in each group). Briefly, CT26^shSTING^ cells (4 × 10^5^ cells/mouse) were suspended in 100 μL of 50% Matrigel and inoculated subcutaneously into the right flank of each mouse. After 10 days, the mice were intraperitoneally injected with 1 × 10^6^ MSCs at 3-day intervals between injections. Irradiation (5 Gy) was performed on Days 11, 16, and 21. The tumor volume was measured every 3 days throughout the study. Tumors were harvested on Day 26 for immunohistochemistry, western blotting, and qRT-PCR. The investigator was blinded to the group allocation of the animals during the experiment. No statistical method was used to predetermine the sample size for the xenograft mice experiment, which was based on previous experimental observations. The sample size of each experiment is shown in the legend. No data were excluded from the analysis.

The longest and shortest diameters (L and W, respectively) of the tumors were measured using Vernier calipers (Sata, Shanghai, China) every 3 days, and the tumor volume (V) was calculated using the formula: V = (L × W^2^)/2. The mice were sacrificed at the termination of the experiments, and the tumor tissues were collected for lysis and subjected to immunoblot analysis and immunohistochemical staining.

### Immunohistochemistry

The antibodies used in this study were as follows: anti-cleaved caspase-3 (#9661, Cell Signaling Technology), anti-mouse CD8a (ab217344, Abcam), anti-Foxp3 (ab215206, Abcam), and anti-mouse granzyme B (ab255598, Abcam). Tissue slides were deparaffinized, incubated with 3% H_2_O_2_ in water for 10 min to quench endogenous peroxidase activity, and subjected to heat mediated antigen retrieval with Antigen Unmasking Solutions (H3300, Vector Laboratories, Burlingame, CA). Tissue sections (3-µm thickness) were stained with the HRP-conjugated avidin-biotin complex (ABC) from the Vectastain Elite ABC Kit (Vector Laboratories, Burlingame, CA) and DAB chromogen (Vector Laboratories) and counterstained with hematoxylin.

Staining for immune cells was positive when detected in the tumor-infiltrating lymphocytes (TILs) and was evaluated using a microscope (OLYMPUS BX53, Tokyo, Japan). Regarding the detection of TILs, the tissue was viewed at 40× magnification, and the area with the highest density of CD8^+^, GzmB^+^, and Foxp3^+^ TILs within the malignant cells was counted at 400× magnification (no. of TILs/high-power field). The average number of tumor-infiltrating immune cells in five high-power fields was included in the evaluation [30].

### Flow cytometry analysis of immune cell profiles

Tumors were dissected from the mice, weighed, and then placed in Petri dishes containing blank RPMI media at room temperature to prevent dehydration. Tumors were minced into small pieces (1–2 mm) with a beaver blade, filtered through a 70-μm strainer, centrifuged, and then resuspended in blank RPMI media. Thereafter, the cell suspensions were layered over Ficoll-Paque media and centrifuged at 1025×*g* for 20 min. The layer of mononuclear cells was transferred into a conical tube, 20 ml of complete RPMI media were added and then gently mixed, and the sample was centrifuged at 650×*g* for 10 min twice. Finally, the supernatant was removed, and the TILs were resuspended in complete RPMI media.

Then, TILs were resuspended in 500 μL of staining buffer (2% BSA and 0.1% NaN_3_ in PBS). The cells were stained with different surface marker panels: (1) CD8^+^ T cells: CD45-PE (E-AB-F1136UD, Elabscience, Texas, USA), CD8a-PerCP (E-AB-F1104UF, Elabscience, Texas, USA); (2) Foxp3^+^ regulatory T cells: CD45-PE (E-AB-F1136UD, Elabscience, Texas, USA), CD4-APC (E-AB-F1097UE, Elabscience, Texas, USA), CD25-PerCP (E-AB-F1194J, Elabscience, Texas, USA), and Foxp3-FITC (E-AB-F1238C, Elabscience, Texas, USA). For intracellular staining, TILs were fixed and permeabilized with Foxp3/transcription factor staining buffer set (eBioscience, Thermo Fisher, CA, USA) after cell-surface stained. Cells were then stained with Foxp3-FITC for 45 min. Samples were washed twice with Perm Wash Buffer and then analyzed by a BD Canto II flow cytometer (BD, CA, USA). Isotype controls were used, including PerCP-conjugated rat IgG2b κ isotype control (E-AB-F09842J), APC-conjugated rat IgG2b (E-AB-F09843E, Elabscience, Texas, USA), and PE-conjugated rat IgG2b κ isotype control (E-AB-F09842D, Elabscience, Texas, USA).

### Treatment of mice with AAV2-sTRAIL-MSCs and PD-L1 blockade

A total of 5 × 10^5^ CT26^shSTING^ cells in 100 μl of 50% Matrigel were inoculated into the right flanks of BALB/c mice. The treatments were initiated on Day 7 after tumor inoculation: AZA (intraperitoneal injection, 0.5 mg/kg/mouse for 3 consecutive days) and 5-FU (intraperitoneal injection, 50 mg/kg/mouse, five times with 3-day intervals between administrations). On Days 9 and 11, mice received radiotherapy (5 Gy), and the PD-L1 inhibitor was administered on Day 11 (100 μg/mouse, intraperitoneal injection, four times with 3-day intervals between injections, Bio×Cell clone 10 F.9G2, NH, USA). The longest and shortest diameters (L and W, respectively) of the tumors were measured using Vernier calipers (Sata, Shanghai, China) every 3 days, and tumor volume (V) was calculated using the following formula: V = (L × W^2^)/2. The mice were sacrificed when the longest diameter reached 20 mm, and the survival of the tumor-bearing mice was observed and recorded every 3 days.

### Tissue microarray (TMA) construction for immunohistochemistry

Colorectal cancer patients who were diagnosed and treated between 2011–2014 at China Medical University Hospital were enrolled in our cohort [30, 31]. The TMA included resected primary tumor tissue and their corresponding normal mucosa specimens, which was approved by Institutional Review Board (IRB) in China Medical University Hospital [Protocol number: CMUH107-REC2–008].

IHC was performed using 3-μm-thick TMA sections with indicated antibodies (anti-human cGAS #79978, Cell Signaling Technology and anti-human STING #13647, Cell Signaling Technology), and then followed with HRP-conjugated avidin-biotin complex (ABC) Kit (Vector Laboratories, CA, USA), incubated with HRP substrate DAB chromogen (Vector Laboratories) and counterstained in hematoxylin [28, 32].

The tumor cGAS and STING staining patterns were evaluated and scored based on the intensity and percentage of positive cells for histoscore (H-score), which was calculated by performing a semiquantitative assessment of both the intensity of the staining (0: negative staining; 1: weak; 2: moderate; and 3: strong staining) and the percentage of immunopositive cells. The H-score ranged from 0 to 300. The expression level was categorized as low or high according to the median value of the H-score [33, 34].

### Statistical analysis

All experiments were conducted at least three times. Statistical analyses were performed using GraphPad Prism 7 statistical software (GraphPad Software, CA, USA) [32]. Data were analyzed using two-way ANOVA followed by Bonferroni’s post hoc test, one-way ANOVA followed by Dunnett’s post hoc test, or an unpaired *t*-test, where appropriate. Data were presented as the mean ± SEM. Student’s *t*-test was used to compare the differences in tumor sizes and positive cell counts between the two groups. ANOVA was used for comparisons of the results involving combinations of MSC, RT, and PD-L1 blockade among the groups. *P* < 0.05 was considered to indicate a significant difference. The survival period was defined as the time from surgery to cancer-specific death, and the cancer-specific survival (CSS) was assessed by Kaplan–Meier survival analysis.

## Results

### Loss of the cGAS/STING axis attenuated RT-induced type I IFN production and suppressed TRAIL-mediated cell death

**Fig. 1:**
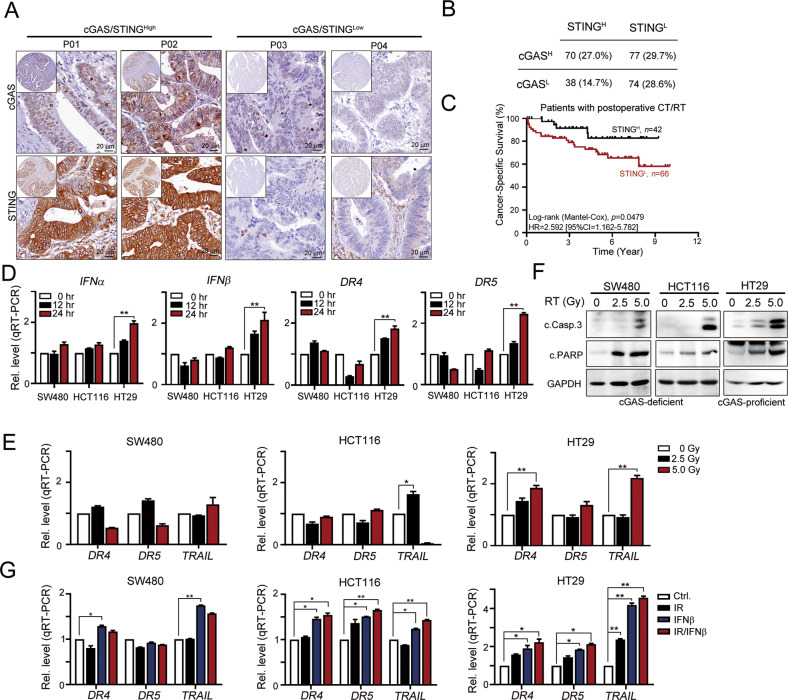
Deficiency of cGAS/STING led to less type I IFN production as well as TRAIL signaling after radiotherapy. **A** The expression of cGAS and STING in colorectal cancer patients (*n* = 259). **B** The deficiency of cGAS and STING was frequently in CRC patients. **C** Low STING1 on tumor cells was associated with poor CSS in colorectal cancer patients who received postoperative chemotherapy and radiotherapy (*n* = 108, Log-rank *p* = 0.0479). CT chemotherapy, RT radiotherapy. **D** SW480, HCT116 (cGAS-deficient), and HT29 (cGAS/STING-proficient) cells were treated with RT (5 Gy) for 12 and 24 h. The mRNA level of *IFNα*, *IFNβ*, TRAIL receptor *DR4* and *DR5* was examined by qRT-PCR (mean ± SEM, *n* = 3). **p < 0.01. **E** SW480, HCT116, and HT29 cells were treated with RT (0, 2.5, and 5 Gy) for 24 h. The mRNA level of *TRAIL*, *DR4*, and *DR5* was examined by qRT-PCR (mean ± SEM, *n* = 3). *p < 0.05 and **p < 0.01. **F** SW480, HCT116, and HT29 cells were treated with RT (0, 2.5, and 5 Gy) for 24 h. The level of caspase-3 and PARP cleavage was examined by western blot. **G** SW480, HCT116, and HT29 cells were treated with RT (5 Gy) and IFNβ (10 ng/mL) for 24 h. The mRNA level of *DR4*, *DR5*, and *TRAIL* was evaluated by qRT-PCR (mean ± SEM, *n* = 3). **p < 0.01.

The cGAS/STING signaling pathway plays critical role in tumor suppression and immune surveillance [35]. Recent studies have shown frequent defects in cGAS/STING-dependent signaling pathways mediated by epigenetic control in several malignancies, including lung cancer and colorectal cancer [16, 18, 36]. By evaluating the tumor levels of cGAS and STING with IHC in a large cohort retrospective study (*n* = 259), we found lower expression levels of cGAS and STING in patients with CRC (*n* = 259, Fig. 1A, B). Most patients with CRC exhibited weak cGAS expression in cancer cells, and 43.2% of patients with CRC lost cGAS expression in cancer cells (112/259 = 43.2%, Fig. 1A). Moreover, 58.3% of patients with CRC did not exhibit STING expression in cancer cells (151/259 = 58.3%, Fig. 1A). Overall, 73% of patients with CRC had defective cGAS/STING signaling (Fig. 1B). Low STING expression on cancer cells was associated with shorter cancer-specific survival (CSS) in patients with CRC who received postoperative DNA-damaging chemotherapy or radiotherapy (Fig. 1C, log-rank *p* = 0.0479, *n* = 108).

**Fig. 2:**
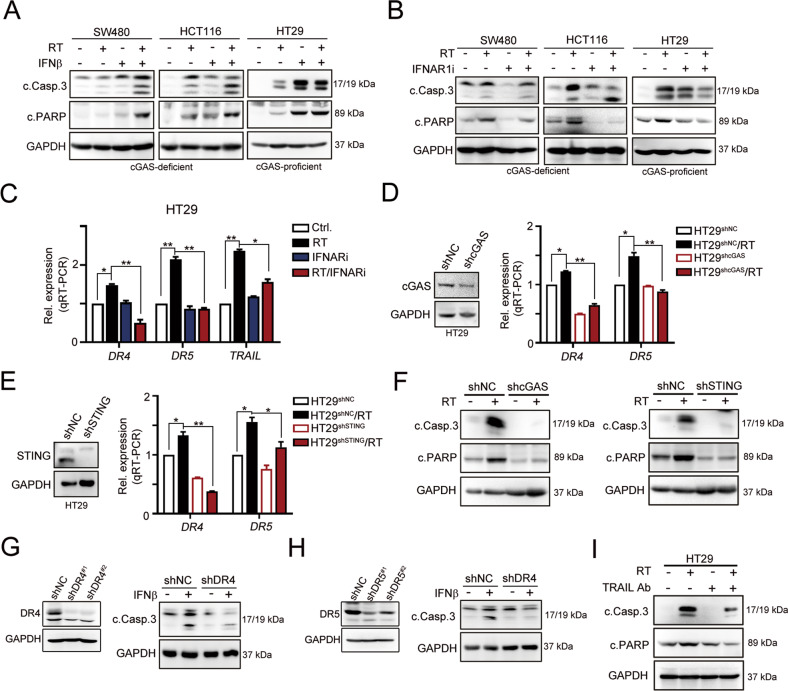
Type I IFN significantly enhanced radiotherapy-induced cell death by TRAIL signaling. **A** Three colorectal cancer cells were treated with IFNβ (10 ng/mL) and RT (5 Gy) for 24 h. The level of caspase-3 and PARP cleavage was evaluated by western blot. **B** Three colorectal cancer cells were treated with IFNAR1 (10 μM) and RT (5 Gy) for 24 h. The level of caspase-3 and PARP cleavage was evaluated by western blot. **C** HT29 (cGAS/STING-proficient) cells were treated with IFNAR1 (10 μM) and RT (5 Gy) for 24 h. The mRNA level of *DR4*, *DR5*, and *TRAIL* was evaluated by qRT-PCR (mean ± SEM, *n* = 3). *p < 0.05 and **p < 0.01. **D** HT29^shNC^ and HT29^shcGAS^ cells were treated with RT (5 Gy). The mRNA level of *DR4*, *DR5*, and *TRAIL* was evaluated by qRT-PCR (mean ± SEM, *n* = 3). *p < 0.05 and **p < 0.01. **E** HT29^shNC^ and HT29^shcGAS^ cells were treated with RT (5 Gy). The mRNA level of *DR4*, *DR5*, and *TRAIL* was evaluated by qRT-PCR (mean ± SEM, *n* = 3). *p < 0.05 and **p < 0.01. **F** HT29^shNC^, HT29^shcGAS^, and HT29^shSTING^ cells were treated with RT (5 Gy). The level of caspase-3 and PARP cleavage was evaluated by western blot. **G** HT29^shNC^ and HT29^shDR4^ cells were treated with IFNβ (10 ng/mL). The level of caspase-3 was evaluated by western blot. **H** HT29^shNC^ and HT29^shDR5^ cells were treated with IFNβ (10 ng/mL). The level of caspase-3 was evaluated by western blot. **I** HT29 cells were treated with TRAIL neutralizing antibodies (1 μg/mL) and RT (5 Gy). The level of caspase-3 and PARP cleavage was evaluated by western blot.

Defective cGAS/STING signaling not only reduced the production of type I IFN for antitumor immunity but also inhibited STING-mediated cell death [12–15, 37], resulting in a poor response to radiotherapy in patients with advanced CRC. STING-dependent cytokines are important mediators of tumor cell killing [37]. By comparing data from patients with CRC who received chemoradiotherapy (GSE15781 dataset), we found that STING-dependent type I IFN and TRAIL signatures were significantly increased in tissues obtained after chemoradiotherapy (CRT) compared with pre-CRT biopsies (Fig. S1A). The level of *IFNβ1* was positively correlated with *TRAIL* expression (Fig. S1B). We analyzed and compared the TRAIL signatures in cGAS-deficient (SW480 and HCT116) and cGAS-proficient (HT29) cells following exposure to radiotherapy to examine whether cGAS/STING-dependent type I IFNs were responsible for TRAIL-mediated cell death (Fig. S1C). We found that the levels of the *IFNα* and *IFNβ* mRNAs were profoundly increased in cGAS-proficient HT29 cells compared to cGAS-deficient SW480 and HCT116 cells (Fig. 1D). Similar results were obtained for the levels of the TRAIL-dependent *DR4* and *DR5* molecules in a time-dependent manner (Fig. 1D). Furthermore, levels of *DR4*, *DR5* and *TRAIL* (Fig. 1E) and the apoptotic indicators cleaved caspase-3 and PARP (Fig. 1F) were significantly increased by radiotherapy in HT29 cells compared to cGAS-deficient SW480 and HCT116 cells (Fig. 1F). Direct addition of IFNβ also increased *DR4*, *DR5* and *TRAIL* expression in these cell lines (Fig. 1G). However, more significant changes in the levels of these molecules were detected in HT29 cells. Combined RT and IFNβ remarkably increased the expression of the *DR4*, *DR5*, and *TRAIL* mRNAs (Fig. 1G). Combination treatment with RT and IFNβ also synergistically increased the cleavage of caspase-3 and PARP (Fig. 2A). Upon IFNAR1 blockade (IFN alpha-IFNAR-IN-1), the cleavage of caspase-3 and PARP was remarkably decreased in cGAS-proficient HT29 cells (Fig. 2B). RT-induced expression of the *DR4*, *DR5*, and *TRAIL* mRNAs was inhibited by the IFNAR1 inhibitor in HT29 cells (Fig. 2C), suggesting that cGAS/STING signaling was involved in RT-induced cell death *via* TRAIL-mediated signaling.

We generated HT29^shcGAS^ and HT29^shSTING^ cells to examine the induction of *DR4*, *DR5*, and *TRAIL* expression by radiotherapy and to verify that the cGAS/STING axis is critical for TRAIL-mediated cell death after treatment with radiotherapy. As shown in Fig. 2D, the levels of the *DR4* and *DR5* mRNAs were significantly decreased in HT29^shcGAS^ cells after RT treatment. The levels of the *DR4* and *DR5* mRNAs were also significantly reduced in HT29^shSTING^ cells after RT treatment (Fig. 2E). Moreover, the cleavage of caspase-3 and PARP was remarkably inhibited in HT29^shcGAS^ and HT29^shSTING^ cells after radiotherapy, respectively (Fig. 2F). Knockdown of TRAIL receptor DR4 and DR5 attenuated IFNβ-mediated cell death (Fig. 2G, H). Blockade of TRAIL by neutralizing antibodies also decreased RT-mediated cell death (Fig. 2I). Based on these results, the cGAS/STING axis was critical for IFNβ-induced cell death via TRAIL.

### Administration of MSCs significantly promotes radiotherapy-induced cell death and delays tumor growth in vivo

**Fig. 3:**
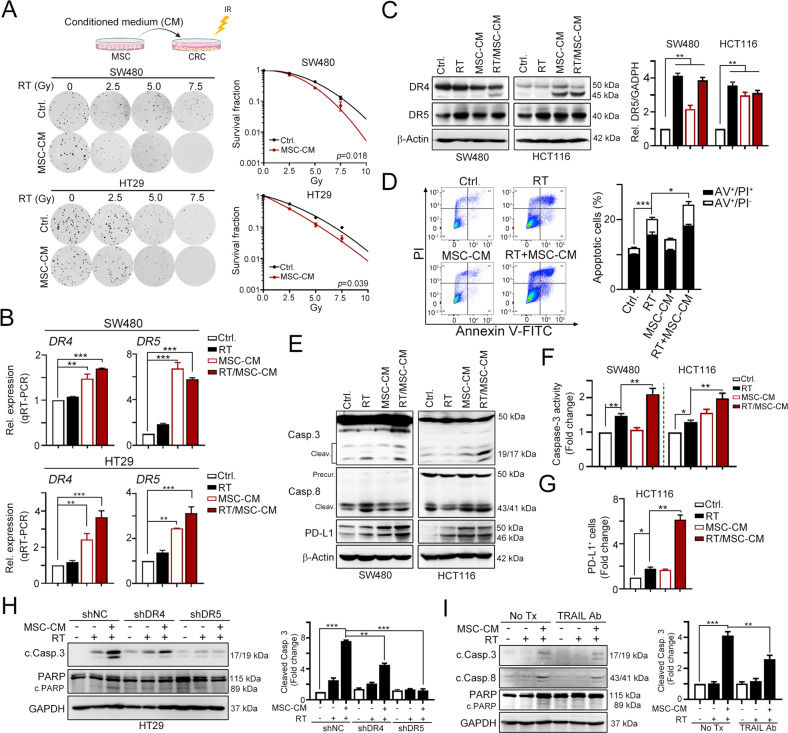
Type I IFN significantly enhanced radiotherapy-induced cell death by TRAIL signaling in cGAS-deficient CRC cells. **A** SW480 and HT29 cells were treated with conditioned medium (CM) from MSCs and RT (5 Gy). After 7 days, the survival fraction was examined by clonogenic assay. **B** SW480 (cGAS-deficient) and HT29 (cGAS/STING-proficient) cells were treated with MSC-CM and RT (5 Gy) for 24 h. The mRNA level of *DR4* and *DR5* was evaluated by qRT-PCR (mean ± SEM, *n* = 3). **p < 0.01 and ***p < 0.001. **C** SW480 and HT29 were treated with MSC-CM and RT (5 Gy) for 24 h. The level of DR4 and DR5 was evaluated by western blot (mean ± SEM, *n* = 3). **p < 0.01 and ***p < 0.001. **D** SW480 cells were treated with MSC-CM and RT (5 Gy) for 24 h. The apoptosis rate was examined by Annexin V-FITC/PI assay (mean ± SEM, *n* = 3). *p < 0.05 and ***p < 0.001. **E** SW480 and HCT116 cells were treated with MSC-CM and RT (5 Gy) for 24 h. The level of caspase-3 and PARP cleavage was evaluated by western blot. **F** SW480 and HCT116 cells were treated with MSC-CM and RT (5 Gy) for 24 h. The activity of caspase-3 was evaluated by a caspase-3 activity assay kit (mean ± SEM, *n* = 3). **p < 0.01. **G** HCT116 cells were treated with MSC-CM and RT (5 Gy). The tumor surface PD-L1 was analyzed by flow cytometry. **H** HT29^shNC^, HT29^shDR4^, and HT29^shDR5^ cells were treated with MSC-CM and RT (5 Gy). The level of caspase-3 and PARP cleavage was evaluated by western blot. **I** HT29 cells were treated with TRAIL neutralizing antibodies (1 μg/mL), MSC-CM and RT (5 Gy). The level of caspase-3 and PARP cleavage was evaluated by western blot.

Several studies have shown that MSCs increase the sensitivity of cells to radiotherapy by inducing TRAIL-dependent cell death [23, 38–40]. Exposure of CRC cells to a conditioned medium from MSCs (MSC-CM) revealed that MSC-CM treatment exerted a significant effect on cancer cell growth and triggered CRC cell death (Fig. S2A, B). Moreover, MSC-CM also increased the sensitivity to radiotherapy, especially in cGAS-deficient SW480 cell lines (Fig. 3A). The levels of the *IFNA1*, *IFNB1*, *DR4*, and *DR5* mRNAs were also significantly increased in both SW480 and HT29 cells treated with MSC-CM (Figs. S2C, 3B). Levels of the DR4 and DR5 proteins were also increased by MSC-CM and RT in SW480 and HCT116 cells (Fig. 3C). Moreover, numbers of apoptotic cells were significantly increased in HCT116 cells (Fig. 3D), indicating that MSC-CM sensitized CRC cells to radiotherapy by inducing TRAIL-dependent cell death, overcoming the cGAS deficiency. The cleavage of caspase-3 and caspase-8 was also significantly increased by MSC-CM/RT (Fig. 3E, F). Moreover, we found that immune checkpoint protein PD-L1 was significantly upregulated by MSC-CM/RT (Fig. 3E, G and Fig. S2D). Knockdown of TRAIL receptors DR4 and DR5 reduced the effect of MSC-CM on RT-mediated cell death (Fig. 3H). Blockade of TRAIL by neutralizing antibodies also showed similar results (Fig. 3I). Taken together, MSCs may increase the response of colorectal cancer cells to radiotherapy via TRAIL signaling.

**Fig. 4:**
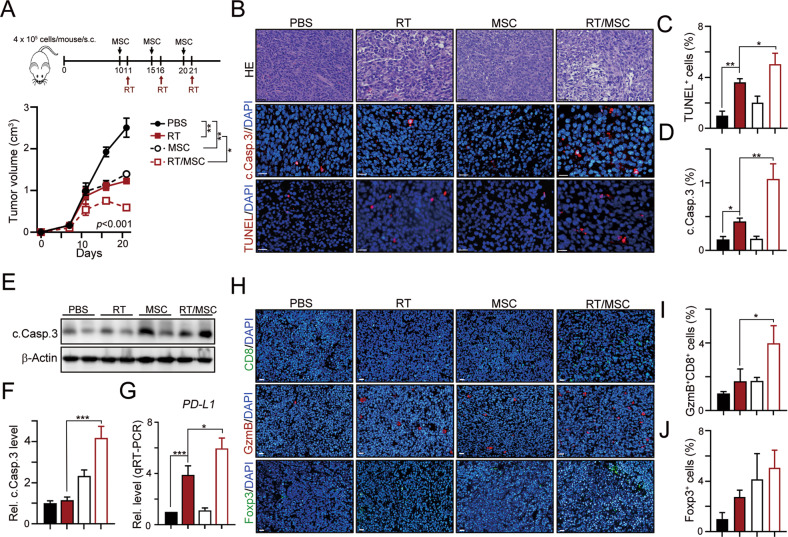
MSC enhanced the therapeuti efficacy of RT by remodulating tumor microenvironment in vivo. **A** About 4 × 10^5^ CT26^shSTING^ cells were subcutaneously inoculated into the left leg of BALB/c mice for 10 days (*n* = 5). About 1 × 10^6^ MSC were intraperitoneally injected into tumor-bearing BALB/c mice on days 10, 15, and 21. Local radiotherapy (5 Gy) was given on days 11, 16, and 21. Tumor volume was measured every 3 days. *p < 0.05 and **p < 0.01. **B** Tumors were harvested on day 26 for immunohistochemistry. The representative result of cleaved caspase-3 and apoptotic cells was shown. Scale bar: 20 μm. **C** The TUNEL^+^ apoptotic cells were evaluated (mean ± SEM, *n* = 3). *p < 0.05 and **p < 0.01. **D** The cleaved caspase-3 cells were evaluated (mean ± SEM, *n* = 3). **p < 0.01 and ***p < 0.001. **E** The level of cleaved caspase-3 was analyzed. **F** The quantification of cleaved caspase-3 cells was shown (mean ± SEM, *n* = 3). ***p < 0.001. **G** The level of *PD-L1 (CD274)* was evaluated by qRT-PCR (mean ± SEM, *n* = 3). ***p < 0.001. **H** The tumor-infiltrating cytotoxic CD8^+^, Granzyme B^+^, and Foxp3^+^ Treg cells was evaluated by immunofluorescent analysis. Scale bar: 20 μm. **I** The quantification of cytotoxic CD8^+^GzmB^+^ cells was shown (mean ± SEM, *n* = 3). *p < 0.05. **J** The quantification of cytotoxic Foxp3^+^ Treg was shown (mean ± SEM, *n* = 3).

By direct administration with MSC-GFP, we found that MSCs significantly homed into tumors after RT treatment, suggesting that MCSs migrated into RT-injured tumor cells (Fig. S3A). Therefore, we subcutaneously inoculated CT26^shSTING^ into the left leg of BALB/c mice and monitored them for 10 days to assess the therapeutic effect of MSCs combined with radiotherapy on CRC by overcoming the cGAS/STING deficiency. Local radiotherapy was administered 1 day after MSCs were injected. The tumor volume also showed a significant decrease in the MSC/RT group (Fig. 4A). Moreover, higher numbers of cleaved caspase-3^+^ and TUNEL^+^ apoptotic cells and higher levels of the cleaved caspase-3 protein in resected tumors were clearly observed in the MSC/RT group compared to the control group (Fig. 4B–F). Based on these results, MSCs enhanced the therapeutic response of STING-deficient colorectal cancer cells to radiotherapy in vivo. Furthermore, we found that PD-L1 expression was elicited in the tumor by MSC-CM (Fig. 3E, F), which is a surrogate for PD1/PD-L1 immunotherapy. Therefore, we analyzed the level of *PD-L1* (*CD274*) in resected tumors using qRT-PCR. We detected high tumor *PD-L1* expression in the MSC/RT group (Fig. 4G). Moreover, the numbers of tumor-infiltrating cytotoxic CD8^+^ and granzyme B^+^ (GzmB^+^) immune cells were significantly increased in the MSC/RT group (Fig. 4H, I and S3B). The infiltration of immunosuppressive Foxp3^+^ T regulatory lymphocytes was not changed (Fig. 4H, J). Taken together, the administration of MSCs not only enhanced the therapeutic efficacy of radiotherapy via TRAIL-mediated cell death but also augmented RT-induced antitumor immunity, suggesting that these cells are suitable for immunotherapy.

### Soluble TRAIL-armed MSCs enhanced the response to immune checkpoint blockade in vivo

**Fig. 5:**
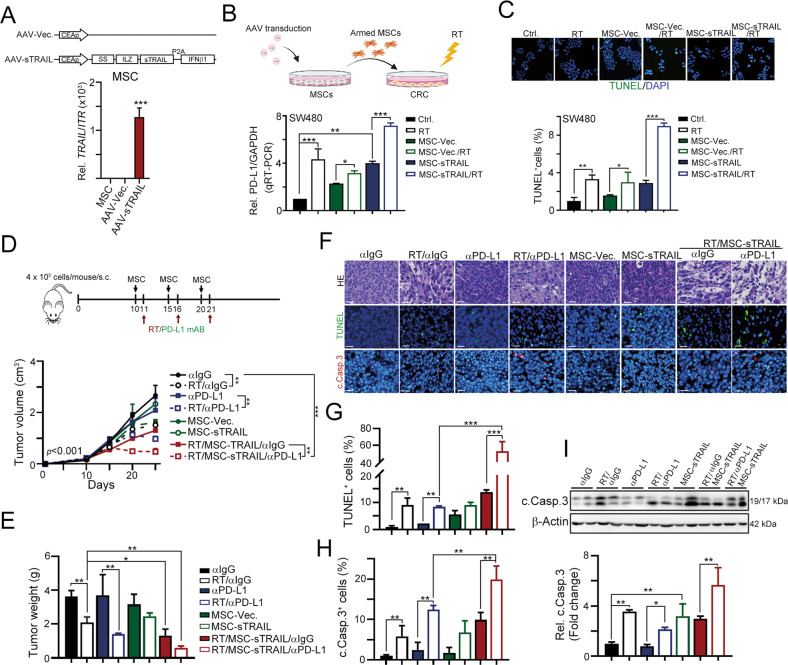
CRC-specific TRAIL-armed MSC significantly enhanced the therapeutic efficacy of radiotherapy and immunotherapy. **A** Schematic depiction of soluble TRAIL (sTRAIL) and IFNβ1 expression cassette. The sTRAIL construct consists of a signal peptide, a Furin cleavage site (Furin CS), an Isoleucine Zipper (ILZ), sTRAIL (aa 114–281) a P2A cleavage site and IFNβ1 sequence. This construct is under the control of the CER promoter within the AAV plasmid. After AAV transduction in MSC, the level of *TRAIL* mRNA was evaluated by qRT-PCR (*n* = 3). ***p < 0.001. **B** SW480 cells (cGAS-deficient) were co-cultured with armed MSCs (MSC-sTRAIL) for 48 h. The level of PD-L1 mRNA was evaluated by qRT-PCR (mean ± SEM, *n* = 3). **p < 0.01 and ***p < 0.001. **C** SW480 cells (cGAS-deficient) were co-cultured with armed MSCs (MSC-sTRAIL) for 48 h. The level of apoptotic cells was evaluated by TUNEL (mean ± SEM, *n* = 3). *p < 0.05, **p < 0.01, and ***p < 0.001. **D** About 4 × 10^5^ CT26^shSTING^ cells were subcutaneously inoculated into the left leg of BALB/c mice for 10 days (*n* = 6–8). About 1 × 10^6^ armed MSC were intraperitoneally injected into tumor-bearing BALB/c mice on days 10, 15, and 21. Local radiotherapy (5 Gy) was given on days 11, 16, and 21. Anti-PD-L1 monoclonal antibodies (100 μg/mouse) and its correspondent IgG were intraperitoneally injected on days 11, 16, and 21. Tumor volume was measured every 3 days. **E** The resected tumors were weighed on day 26 (mean ± SEM, *n* = 5). **F** The cleaved caspase-3 cells and apoptotic cells were analyzed by immunohistochemistry (mean ± SEM, *n* = 3). The representative result of cleaved caspase-3 and apoptotic cells was shown. Scale bar: 20 μm. **G** The TUNEL^+^ apoptotic cells were evaluated (mean ± SEM, *n* = 3). **p < 0.01 and ***p < 0.001. **H** The cleaved caspase-3 cells were evaluated (mean ± SEM, *n* = 3). **p < 0.01. **I** The level of cleaved caspase-3 was analyzed by western blot.

Due to the tumor-homing property of MSCs, MSCs armed to deliver soluble TRAIL were reported to enhance the therapeutic efficacy of chemotherapy, HDAC inhibitors and radiotherapy in several animal models [40–42]. Therefore, we engineered adenovirus-associated virus (AAV) with a soluble form (sTRAIL, aa 114–281) and IFNB1 fused with an isoleucine zipper (ILZ) for trimerization, a signal peptide of the human fibrillin gene that ensures effective secretion and a furin cleavage site to release the ILZ-sTRAIL protein into the extracellular space and a P2A cleavage site to maintain IFNB1 in the cytoplasm under the control of the CRC-specific CEA promoter (Fig. 5A). The level of the *TRAIL* mRNA was significantly increased in MSCs after AAV transduction (Fig. 5A). The conditioned medium (CM) of sTRAIL-armed MSCs (MSC-sTRAIL) alone substantially increased the levels of *PD-L1, DR4*, and *DR5* in HCT116 cells (Fig. 5B and Fig. S4A, B). The CM from MSC-sTRAIL also significantly increased RT-induced cell death (Fig. 5C). Moreover, the effect was significantly enhanced by RT (Fig. 5B, C and Fig. S4C) and remarkably decreased in DR4 or DR5-deficient cells (Fig. S4D, E), suggesting that the armed MSCs were superior to sensitize STING-deficient cells to radiotherapy via TRAIL receptor.

Since our results showed that higher PD-L1 levels were elicited by MSC-sTRAIL, we evaluated whether MST-sTRAIL enhanced the therapeutic efficacy of radiotherapy and immunotherapy in STING-deficient CRC cells. Ten days after the subcutaneous inoculation of CT26^shSTING^ cells into the left leg of BALB/c mice, MSCs carrying sTRAIL were intraperitoneally injected at 5-day intervals. Local radiotherapy (5 Gy) and anti-PD-L1 (100 μg/mouse) were administered one day after the MSC injection (Fig. 5D). As shown in Fig. 5D, we found that the tumor volume was substantially reduced when MSC-sTRAIL were administered in combination with RT. However, a large extent of tumor regression was observed in the RT/MSC-sTRAIL/anti-PD-L1 group. The resected tumor weight was also reduced in the RT/MSC-sTRAIL/anti-PD-L1 group on Day 26 (Fig. 5E). The numbers of TUNEL^+^ and cleaved caspase-3^+^ apoptotic cells were remarkably increased in the RT/MSC-sTRAIL/anti-PD-L1 group (Fig. 5F–H). Cleaved caspase-3 was also clearly detected (Fig. 5I). Taken together, these results showed that MSC-sTRAIL synergistically increased the therapeutic efficacy of radiotherapy and immunotherapy by overcoming the deficiency in cGAS/STING signaling.

### sTRAIL-armed MSCs remodeled the tumor microenvironment by increasing the infiltration of immune cells

**Fig. 6:**
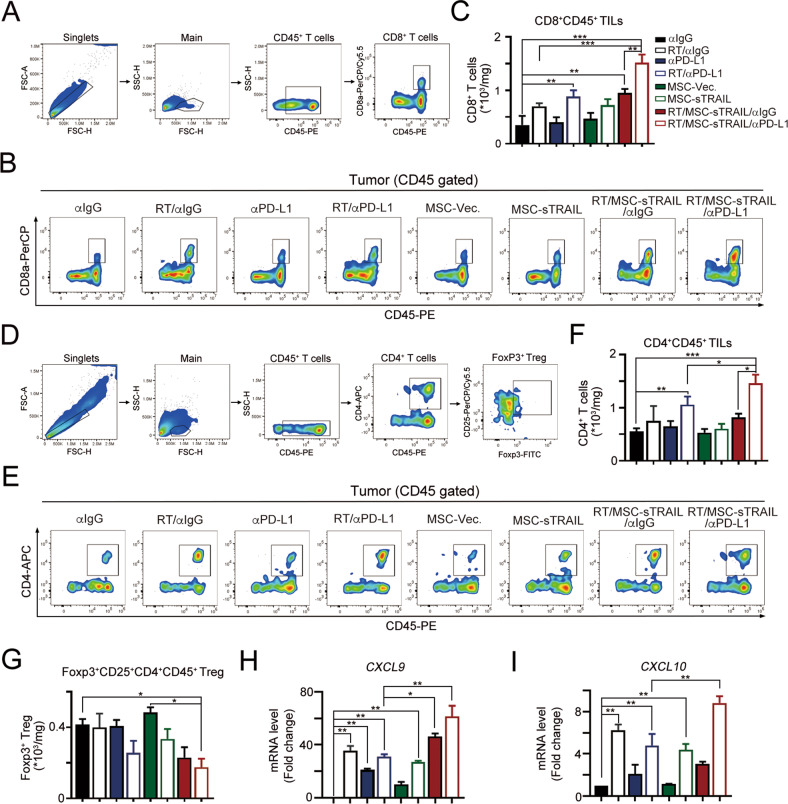
Armed MSC significantly increased the tumor-infiltrating CD4 and CD8 after radiotherapy and immunotherapy in vivo. **A** The gating strategy for tumor-infiltrating CD8. **B** The representative image of tumor-infiltrating CD8 cells. **C** The number of tumor-infiltrating CD8 cells within resected tumors was evaluated by flow cytometry (mean ± SEM, *n* = 3). ***p < 0.001. **D** The gating strategy for tumor-infiltrating CD4 and Foxp3^+^ regulatory T cells. **E** The representative image of tumor-infiltrating CD4 cells. **F** The number of tumor-infiltrating CD4 cells within resected tumors was evaluated by flow cytometry (mean ± SEM, *n* = 3). ***p < 0.001. **G** The tumor-infiltrating Foxp3^+^ Treg cells within resected tumors was evaluated by flow cytometry (mean ± SEM, *n* = 3). *p < 0.05 and **p < 0.01. **H** The level of *CXCL9* mRNA within the tumor microenvironment was evaluated by qRT-PCR (mean ± SEM, *n* = 3). *p < 0.05 and **p < 0.01. **I** The level of *CXCL10* mRNA within the tumor microenvironment was evaluated by qRT-PCR (mean ± SEM, *n* = 3). *p < 0.05 and **p < 0.01.

Tumor-infiltrating lymphocytes were isolated for further analysis to further evaluate whether sTRAIL-armed MSCs increased the therapeutic efficacy of radiotherapy and immunotherapy by remodeling the tumor microenvironment (Fig. 6A). Using flow cytometry, we found that the density of tumor-infiltrating CD8^+^ cells was only slightly changed in animals injected with MSC-sTRAIL (Fig. 6B, C). However, MSC-sTRAIL significantly increased the density of CD8^+^ TILs when combined with RT and immunotherapy (Fig. 6B, C). A significant change in the number of tumor-infiltrating CD4^+^ cells was also observed in the RT/MSC-sTRAIL/anti-PD-L1 group (Fig. 6D–F). There is slightly increase in the density of immunosuppressive Foxp3^+^ Tregs when administrated with MSC-Vec. alone (Fig. 6G). But the infiltration of Foxp3^+^ Tregs was significantly decreased in the RT/MSC-sTRAIL/anti-PD-L1 group (Fig. 6G). Furthermore, the proinflammatory cytokines such as *CXCL9* and *CXCL10* were significantly increased in RT/MSC-sTRAIL/anti-PD-L1 group (Fig. 6H, I). Taken together, these results showed that the administration of MSC-sTRAIL remodeled the tumor microenvironment by upregulating proinflammatory cytokine production as well as increasing the infiltration of immune cells to enhance the therapeutic efficacy of radiotherapy and immunotherapy.

## Discussion

Radiotherapy is an essential therapeutic option for a wide range of tumor types, including colorectal cancer. However, its therapeutic effect and long-term survival varied according to the extent of antitumor immunity. In the present study, we found that RT-activated cGAS/STING signaling not only promoted type I IFN expression for antitumor immunity but also triggered TRAIL-dependent cell death. Administration of MSCs enhanced the therapeutic effect of radiotherapy by inducing type I IFN expression to activate the TRAIL signaling pathway. The molecular mechanism underlying MSC-mediated radiosensitivity in STING-deficient CRC cells may be to overcome defective cGAS/STING-mediated antitumor immunity and TRAIL-dependent cell death. Moreover, the therapeutic efficacy was significantly enhanced by armed MSCs with secreted TRAIL and IFNβ1. In addition, the administration of armed MSCs also remodeled the tumor microenvironment for a better response to immunotherapy in a STING-deficient CRC animal model. Notably, we revealed that the cGAS/STING pathway affects antitumor immunity and cellular radiosensitivity, which can be reactivated by the factors secreted by armed MSCs. Taken together, MSCs functioned as tumor-specific carriers for the development of immunotherapeutic agents for immunodeficient patients with colorectal cancer.

Accumulating evidence has shown that MSCs mediate the suppression of cancer cell proliferation [21, 22, 27, 43–45], indicating that MSCs inhibit the growth and promote the apoptosis of cancer cells. Direct administration of MSCs significantly inhibited the PI3K/AKT signaling pathway [45], Stat3 signaling pathway [27], and VEGF expression [46]. The miRNAs carried in MSC-derived exosomes have been reported to inhibit the growth and progression of multiple malignancies, such as hepatocellular carcinoma (HCC) [47] and pancreatic cancer [26]. Moreover, MSCs also enhanced the therapeutic efficacy of radiotherapy in breast cancer, colorectal cancer, and HCC [20, 25, 27]. Consistent with these studies, our findings showed that MSCs enhanced the effect of radiotherapy on tumors by inhibiting tumor cell proliferation and enhancing TRAIL-dependent cell death by promoting cGAS/STING-mediated type I production. We showed that MSC-CM inhibited colorectal cancer cell proliferation and TRAIL-mediated apoptosis. Furthermore, MSC-CM increased RT-induced cell death, especially in STING-deficient colorectal cancer cells. Most patients with CRC in our cohort were deficient in either cGAS or STING. Approximately 60% of patients with CRC lose STING expression, which might be suppressed by methylation [48]. RT-induced dsDNA fragments are recognized by cGAS to trigger STING-mediated production of type I IFN and several proinflammatory cytokines for anticancer immunity [5–8]. Recently, innate immune sensing by dendritic cells following radiotherapy was shown to be dominated by the cGAS/STING-dependent pathway, which drives the adaptive immune response to ionizing radiation [8–10]. In addition to antitumor immunity, the cGAS/STING pathway is also associated with multiple functions, including cell death. cGAS and STING promote mitotic cell death and ER stress-associated cell death [49, 50]. More recently, Hayman et al. identified that STING directly increases cell death by regulating radiotherapy-induced reactive oxygen species production and DNA damage in individuals with head and neck squamous cell carcinoma [51]. Downstream IFNα/β signaling has been shown to trigger TRAIL expression and subsequent cell death in breast cancer and bladder cancer [14, 15, 52, 53]. Defects in cGAS and STING may not only attenuate antitumor immunity but also reduce the extent of type I IFN-dependent cell death. Here, we showed that MSCs may release TRAIL to increase the sensitivity of STING-deficient colorectal cancer to RT. Moreover, MSCs armed with CRC-specific TRAIL significantly increased the sensitivity to RT and remodeled the tumor microenvironment for immunotherapy. Consistent with our findings, Francois et al. found that the administration of MSCs significantly attenuates colon cancer progression by modulating the tumor microenvironment [54]. They found that MSCs activate an anti-inflammatory response by polarizing resident macrophages into anti-inflammatory macrophages to inhibit tumor initiation. Furthermore, Feng et al. showed that MSC administration combined with radiotherapy promoted cell death and inhibited PI3K/Akt signaling in CRC [20]. Based on these results, MSCs are capable of enhancing the therapeutic efficacy of radiotherapy through multiple mechanisms. Moreover, several studies reported that MSC-derived exosomes inhibit cancer cell proliferation, invasion, and migration through the microRNAs they carry, such as miRNA-15a [47] and miRNA-126-3p [26]. Therefore, the detailed mechanisms by which MSCs increase the sensitivity to RT and remodel the tumor microenvironment require further elucidation.

In addition to the direct effect of MSCs on cancer cell progression, several groups have developed armed MSCs for cancer gene therapy due to their tumor tropism properties. The therapeutic potential of stem cell-based gene therapy using MSCs expressing sTRAIL has been documented in different preclinical models [40, 42, 55, 56]. For safety and tumor specificity, we developed a colorectal cancer-specific system controlled by the CEA promoter in the AAV system. MSC-sTRAIL significantly increased the responses to radiotherapy and immunotherapy. Supporting our findings, armed MSC-sTRAIL enhance the therapeutic response to paclitaxel and AKTi in PDAC [41, 56], HDAC inhibitors in glioma [42], radiotherapy [20, 23, 25, 27, 39, 57, 58] and immunotherapy [59]. Many studies have indicated that TRAIL-sensitizing strategies combining TRAIL with chemotherapeutic agents and radiotherapy exert enhanced therapeutic effects by simultaneously targeting multiple mechanisms, thus requiring a lower dose to prevent adverse side effects [60]. Furthermore, an elegant study by Zhang et al. showed that patients with deficient type I IFN signaling were associated with poor survival outcomes in melanoma [61]. Administration with IFNα-modified MSCs empowers T cells to enhance antitumor immunity and increase the response to PD-L1 blockade by the negative feedback mechanism of PD-L1 upregulation [61]. They found that IFNα-MSCs increased the cytotoxic ability of T cells by STAT3-dependent GzmB upregulation. Consistent with their finding, our results showed that MSC-sTRAIL elicited therapeutic efficacy of radiotherapy by recruiting more infiltration of cytotoxic T cells, especially when combined with PD-L1 blockade. Similarly, PD-L1 upregulation might be triggered by TRAIL as well as IFNβ to increase the response to PD-L1 [11]. Therefore, our results showed that dual IFNβ- and sTRAIL-modified MSCs may provide high therapeutic efficacy by either direct cell killing or indirect antitumor immunity, suggesting that the use of MSCs for tumor-specific gene therapy is a potential therapeutic strategy for cancer treatment.

Taken together, our studies showed that MSCs armed with soluble TRAIL provided higher therapeutic efficacy when combined with radiotherapy and immunotherapy. These strategies overcome the low antitumor immunity elicited by RT in cGAS/STING-deficient colorectal cancer.

## Supplementary information

Supplemental information

Reproducibility checklist

Western blot original data

## Data Availability

The original dataset is available on request from the corresponding author.
