# Supplementary material for: Engineered sTRAIL-armed MSCs overcome STING deficiency to enhance the therapeutic efficacy of radiotherapy for immune checkpoint blockade
Source: Cell Death Dis. 2022 Jul 14;13(7):610. doi: 10.1038/s41419-022-05069-0 (PMC9283452; doi:10.1038/s41419-022-05069-0)
Supplement: Supplementary file 1 — Supplemental information [file 41419_2022_5069_MOESM1_ESM.docx]

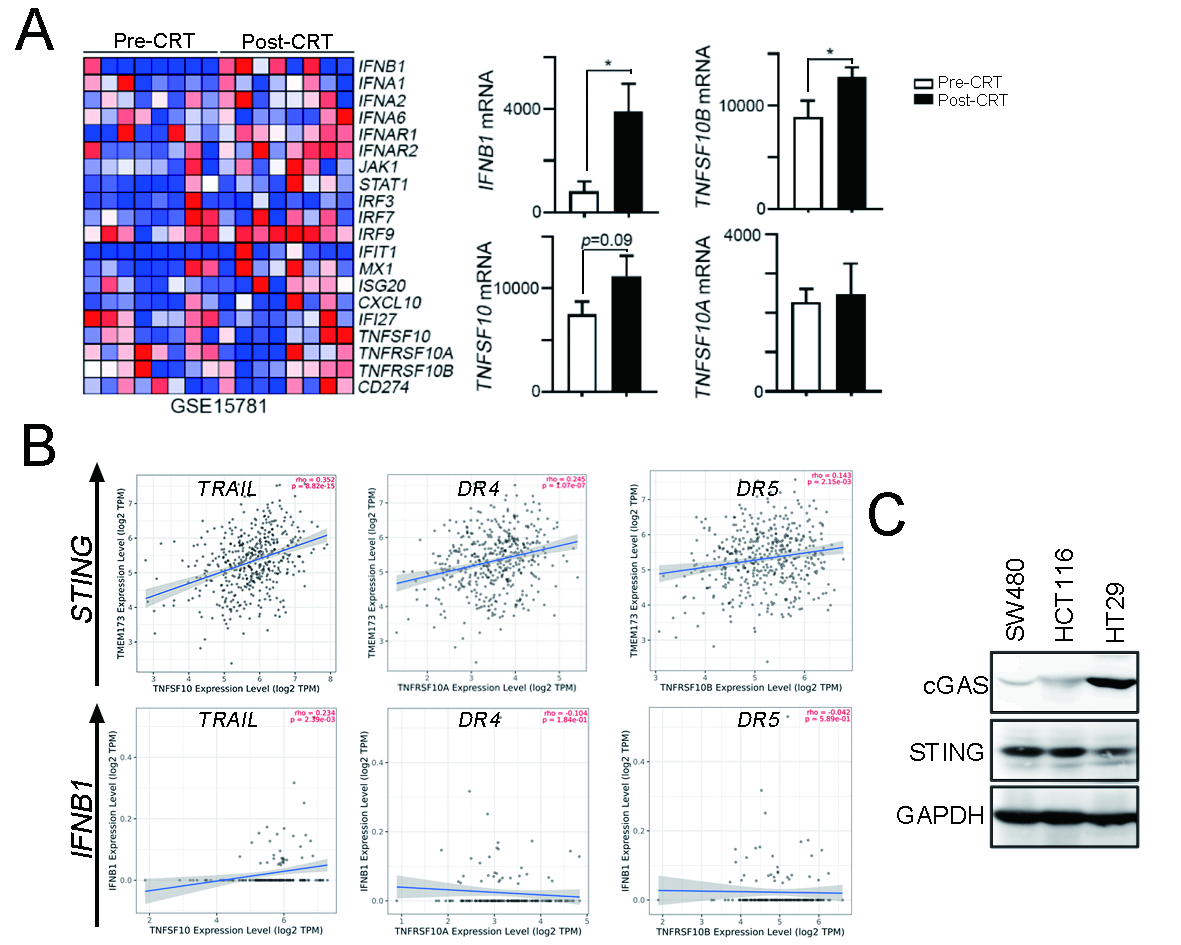


**Figure S1. Type I IFN and TRAIL signaling was elevated by chemoradiotherapy treatment.**

1. The mRNA data were retrieved from NCBI (GSE93375 database). The type I IFN signatures and TRAIL signaling were analyzed for heatmap (pre-CRT:8 CRC patients; post-CRT:8 CRC patients).
2. The level of *STING1* and *IFNB1* was positively correlated with *TRAIL* expression (*p*=0.00239, *r*=0.234). There is not significant correlation between *DR4*, *DR5* and *IFNB1.*
3. The level of cGAS and STING in colorectal cancer.

**
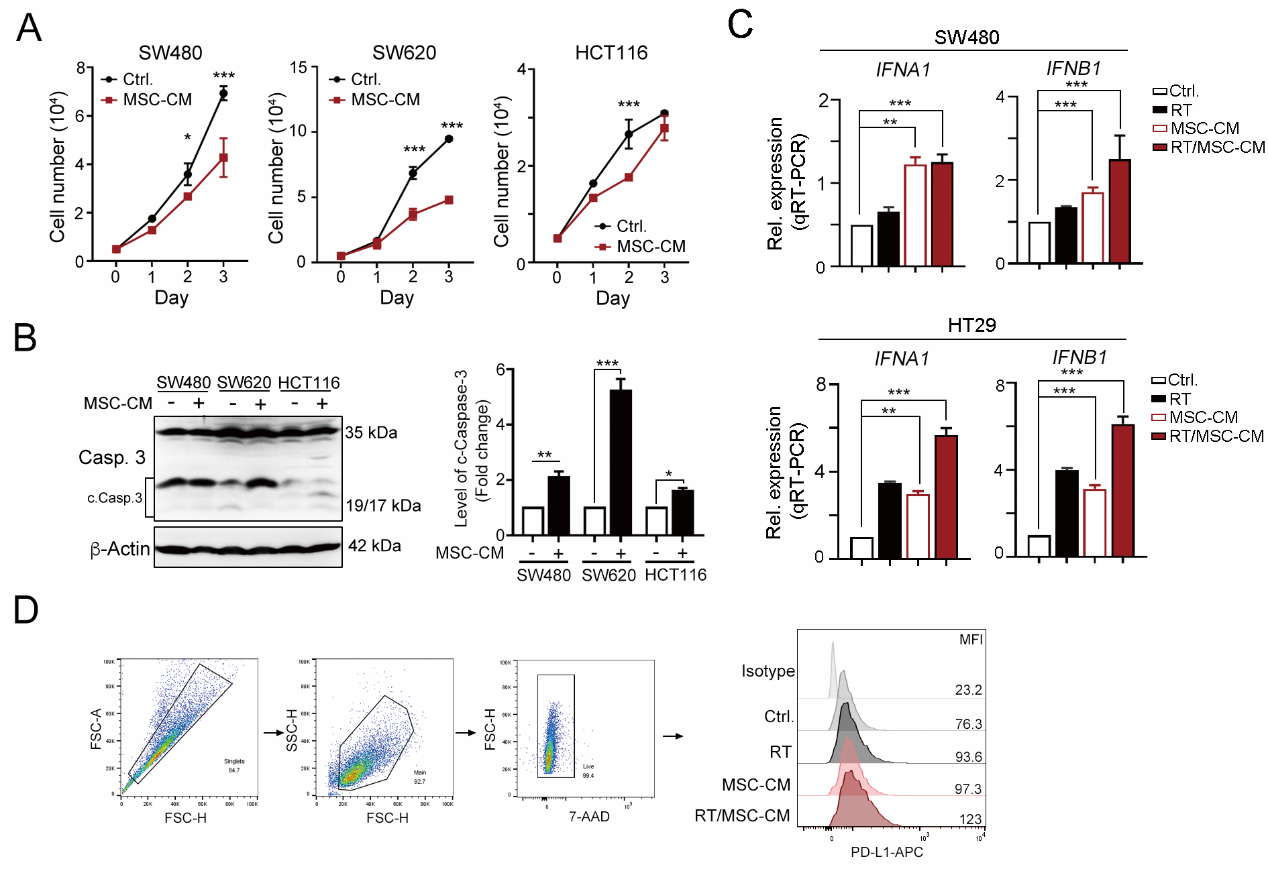
Figure S2. Conditioned medium from MSC inhibited cell growth, induced apoptosis and promoted RT-induced type I IFN production.**

1. The MSC-CM significantly inhibited cell growth in colorectal cancer cell.
2. Addition of MSC-CM enhanced caspase-3-mediated cell death (n=3). **p*<0.05, ***p*<0.01 and ****p*<0.001.
3. Addition of MSC-CM remarkably triggered type IFN production. Moreover, MSC-CM significantly increased the effect of RT for type I IFN production (n=3). ***p*<0.01 and ****p*<0.001.
4. The gating strategy of flow cytometry to evaluate surface PD-L1. HCT116 cells were treated with MSC-CM and RT (5Gy). The tumor surface PD-L1 was analyzed by flow cytometry.


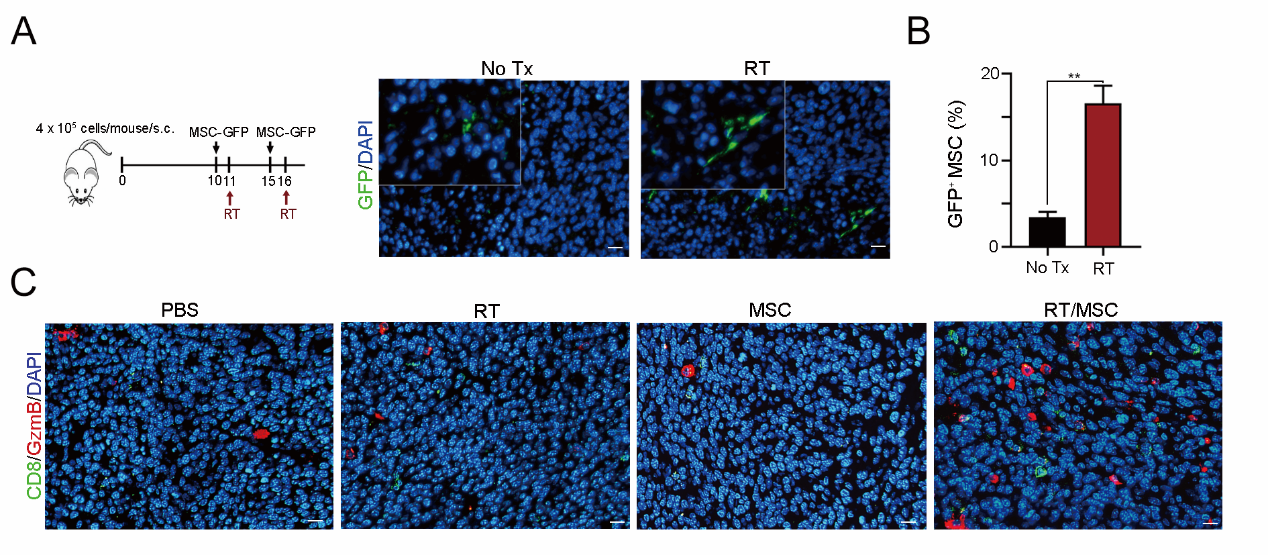


**Figure S3. Intraperitoneal administrated MSCs migrated into tumor lesion and attracted immune cell infiltration.**

1. 4×10^5^ CT26 cells were subcatanous inoculated into left leg of BALB/c mice for 10 days (n=5). 1×10^6^ MSC-GFP cells were intraperitonal injected into tumor-bearing BALB/c mice on day 10 and 15. Local radiotherapy (5 Gy) was given on day 11, and 16. Tumors were harvest on day 20 for immunofluorescent analysis.
2. MSC-GFP cells were significantly increased after radiotherapy.
3. The tumor-infiltrating CD8^+^GzmB^+^ cells were evaluated by immunofluorescent analysis. The statistical results were shown in Fig. 4I. Scale bar: 20 μm.


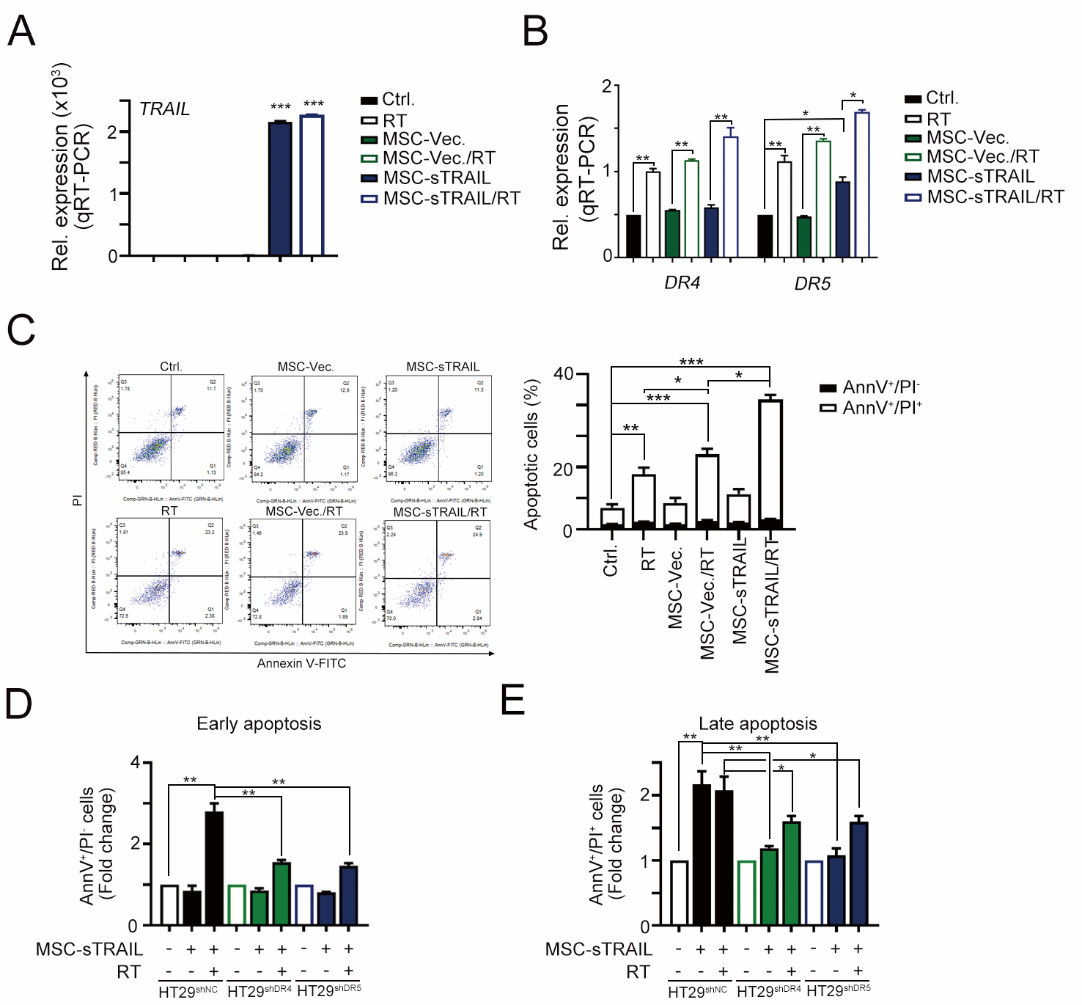


**Figure S4. Armed MSC inhibited cell growth and induced apoptosis.**

1. The level of TRAIL in MSCs after AAV transduction was evaluated by qRT-PCR.
2. Armed MSC also enhanced RT-induced *DR4* and *DR5* expression (n=3). **p*<0.05, ***p*<0.01 and ****p*<0.001.
3. Addition of CM from MSC-TRAIL also significantly increased the effect of RT for apoptosis (n=3). **p*<0.05, ***p*<0.01 and ****p*<0.001.
4. The early apoptotic cells in HT29^shNC^, HT29^shDR4^ and HT29^shDR5^ after treatments with MSC-TRAIL conditioned medium and RT were evaluated by flow cytometry.
5. The late apoptotic cells in HT29^shNC^, HT29^shDR4^ and HT29^shDR5^ after treatments with MSC-TRAIL conditioned medium and RT were evaluated by flow cytometry.

| **Table S1. Clinicopathological parameters of colorectal cancer patients (n=259)** | | | | | | | | |
| --- | --- | --- | --- | --- | --- | --- | --- | --- |
| Clinicopathological parameters | Total no. | **Tumor cGAS** | | *p* value |  | **Tumor STING** | | *p* value |
|  |  | Low | High |  |  | Low | High |  |
|  | 259 | 112 | 147 |  |  | 151 | 108 |  |
| Gender |  |  |  | 0.675 |  |  |  | 0.283 |
| Female | 98 | 44 | 54 |  |  | 53 | 45 |  |
| Male | 161 | 68 | 93 |  |  | 98 | 63 |  |
| Age |  |  |  | 0.365 |  |  |  | 0.086 |
| <65 | 142 | 65 | 77 |  |  | 76 | 66 |  |
| ≥65 | 117 | 46 | 70 |  |  | 75 | 42 |  |
| pT stage |  |  |  | 0.368 |  |  |  | 0.856 |
| pT1 | 26 | 15 | 11 |  |  | 14 | 12 |  |
| pT2 | 56 | 26 | 30 |  |  | 32 | 24 |  |
| pT3 | 149 | 60 | 89 |  |  | 90 | 59 |  |
| pT4 | 28 | 11 | 17 |  |  | 15 | 13 |  |
| pN stage |  |  |  | 0.339 |  |  |  | 0.078 |
| N0 | 116 | 53 | 63 |  |  | 70 | 46 |  |
| N1 | 86 | 39 | 47 |  |  | 55 | 31 |  |
| N2 | 57 | 20 | 37 |  |  | 26 | 31 |  |
| TNM stage (7^th^ AJCC) |  |  |  | 0.154 |  |  |  | 0.859 |
| Stage I | 58 | 30 | 28 |  |  | 34 | 24 |  |
| Stage II | 54 | 23 | 31 |  |  | 34 | 20 |  |
| Stage III | 111 | 49 | 62 |  |  | 62 | 49 |  |
| Stage IV | 36 | 10 | 26 |  |  | 21 | 15 |  |
| Lymphovascular invasion |  |  |  | 0.776 |  |  |  | 0.158 |
| Absent | 157 | 69 | 88 |  |  | 97 | 60 |  |
| Present | 102 | 43 | 59 |  |  | 54 | 48 |  |
| Perineural invasion |  |  |  | 0.292 |  |  |  | 0.158 |
| Absent | 157 | 72 | 85 |  |  | 97 | 60 |  |
| Present | 102 | 40 | 62 |  |  | 54 | 48 |  |
| Pearson test was used, and Fisher's exact test was used when counts were less than 5. The test did not include the “unknown or unspecified" group. Only stage I-III patients were included in the statistical analysis on LR, DM and tumor relapse. | | | | | | | | |
