# Supplementary material for: Engineered sTRAIL-armed MSCs overcome STING deficiency to enhance the therapeutic efficacy of radiotherapy for immune checkpoint blockade
Source: Cell Death Dis. 2022 Jul 14;13(7):610. doi: 10.1038/s41419-022-05069-0 (PMC9283452; doi:10.1038/s41419-022-05069-0)
Supplement: Supplementary file 3 — Western blot original data [file 41419_2022_5069_MOESM3_ESM.pdf]

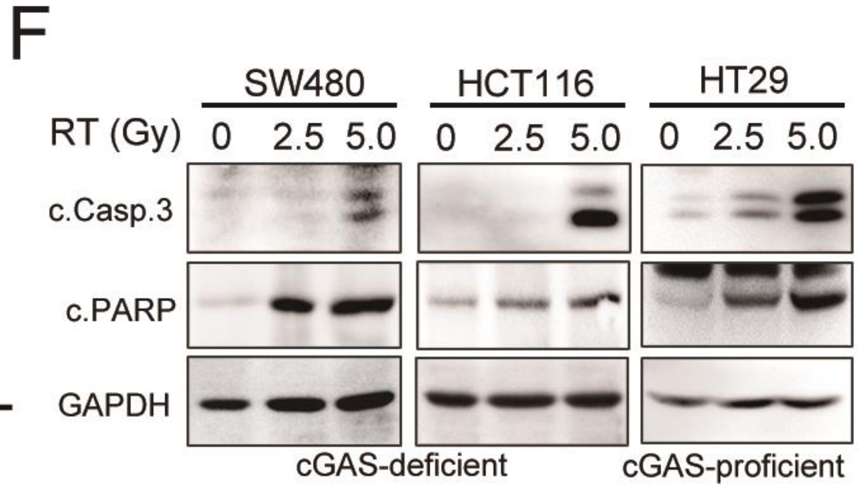
F
SW480
HCT116
HT29
RT (Gy)
0 2.5 5.0
0 2.5 5.0
0 2.5 5.0
c.Casp.3
c.PARP
GAPDH
cGAS-deficient
cGAS-proficient

Fig. 1F

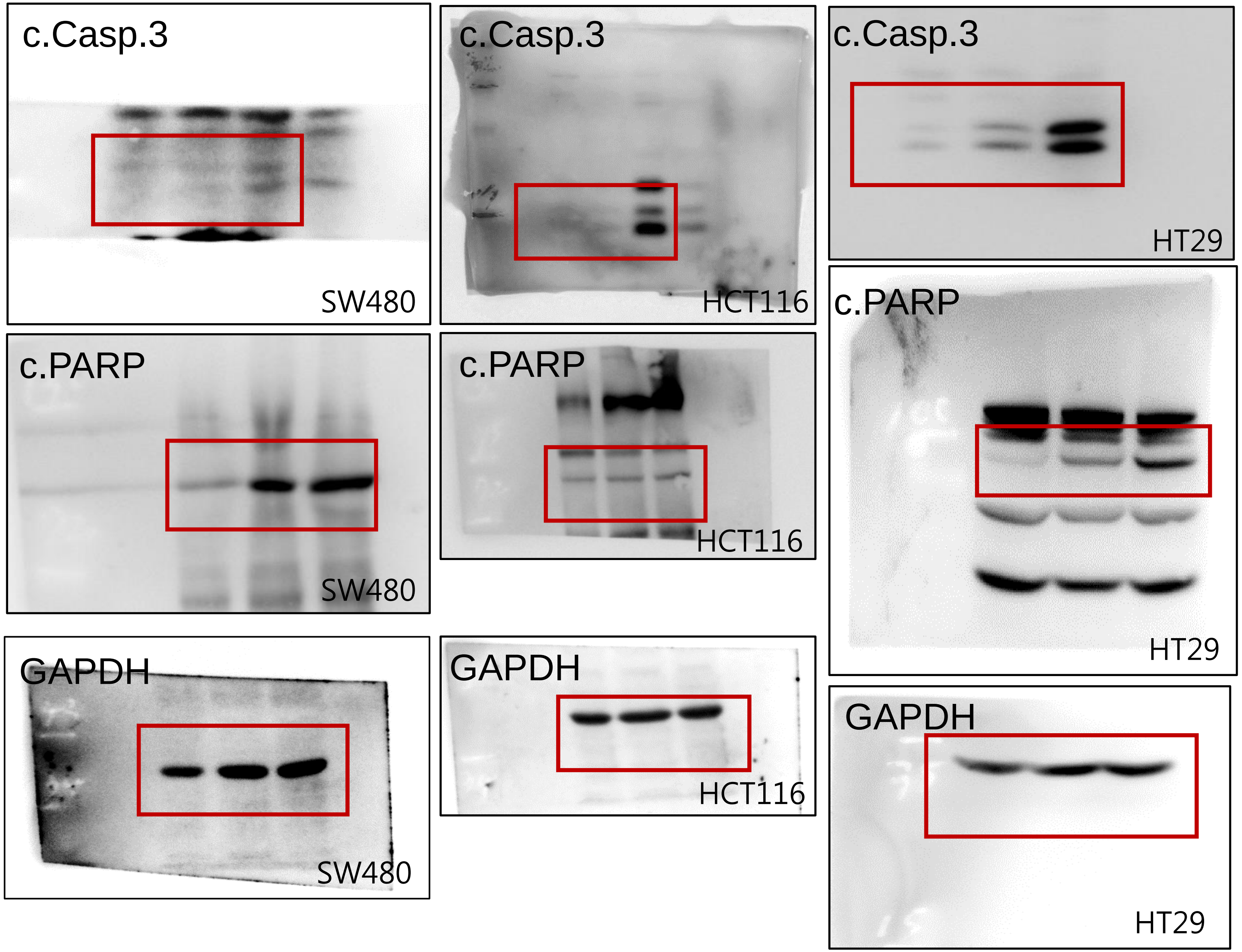
c.Casp.3
SW480
c.Casp.3
HCT116
c.Casp.3
HT29
c.PARP
SW480
c.PARP
HCT116
c.PARP
HT29
GAPDH
SW480
GAPDH
HCT116
GAPDH
HT29

A

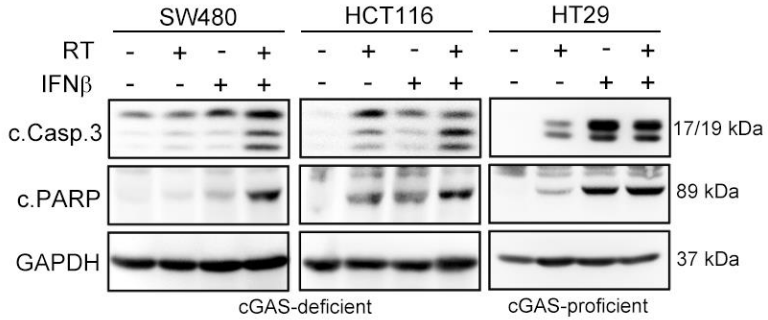
SW480
HCT116
HT29
RT - + - + - + - + - + - +
IFNβ - - + + - - + + - - + +
c.Casp.3
17/19 kDa
c.PARP
89 kDa
GAPDH
37 kDa
cGAS-deficient
cGAS-proficient

Fig. 2A

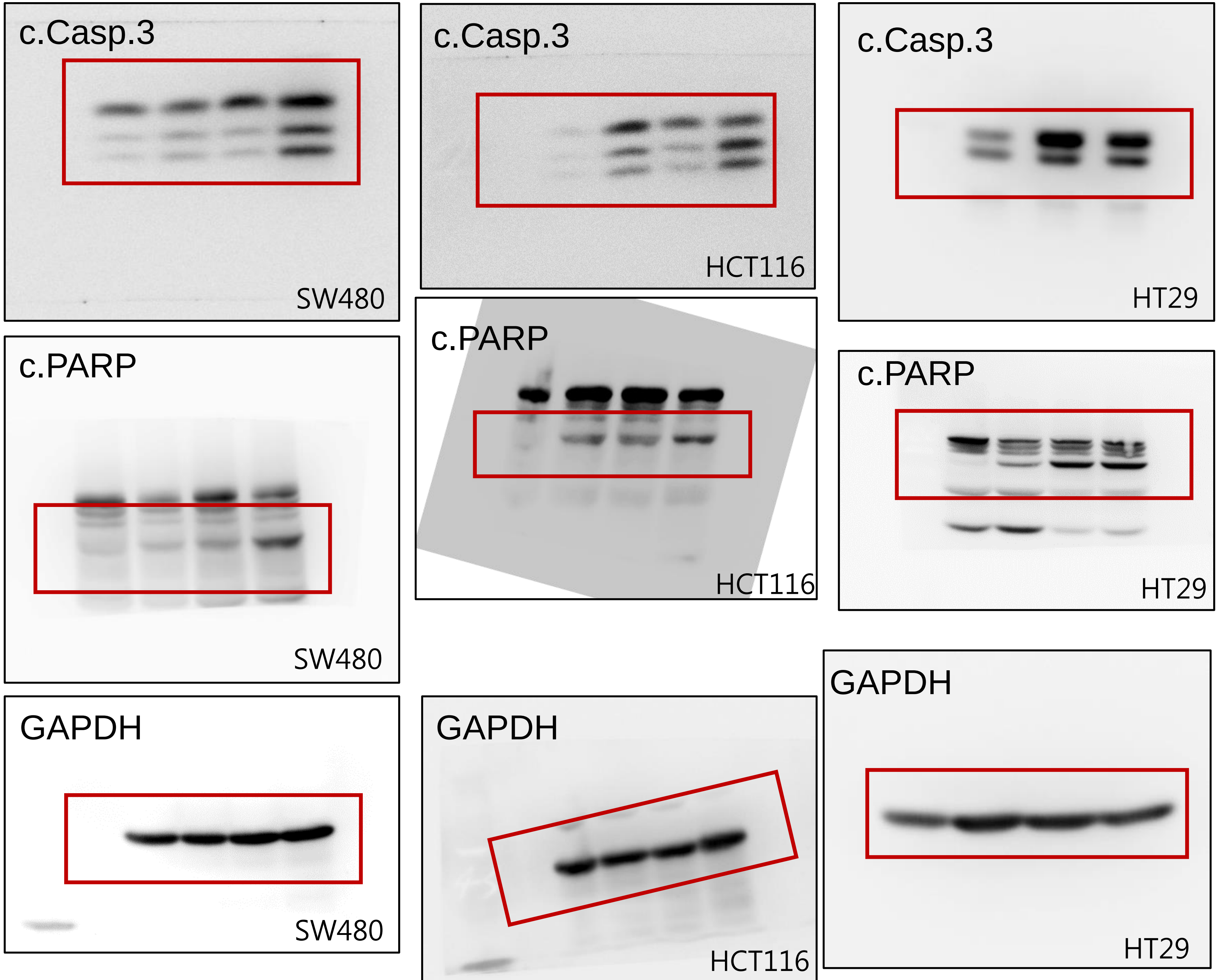
c.Casp.3
SW480
c.Casp.3
HCT116
c.Casp.3
HT29
c.PARP
SW480
c.PARP
HCT116
c.PARP
HT29
GAPDH
SW480
GAPDH
HCT116
GAPDH
HT29

B

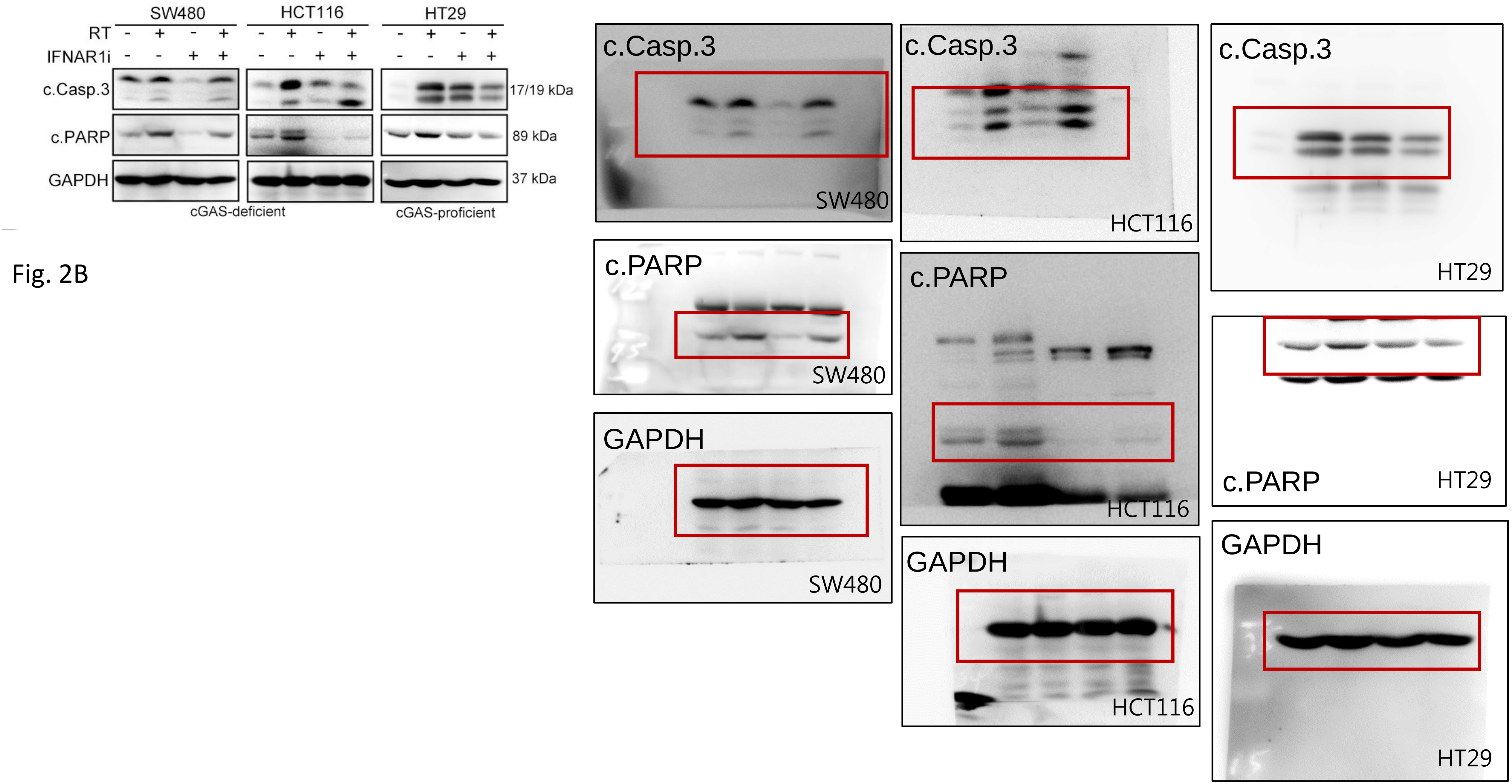
SW480
HCT116
HT29
RT - + - + - + - + - + - +
IFNAR1i - - + + - - + + - - + +
c.Casp.3
c.PARP
GAPDH
17/19 kDa
89 kDa
37 kDa
cGAS-deficient
cGAS-proficient
c.Casp.3
SW480
c.Casp.3
HCT116
c.Casp.3
HT29
c.PARP
SW480
c.PARP
HCT116
c.PARP
HT29
GAPDH
SW480
GAPDH
HCT116
GAPDH
HT29

Fig. 2B

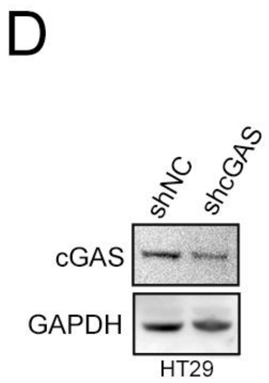
D
shNC
shcGAS
cGAS
GAPDH
HT29

Fig. 2D

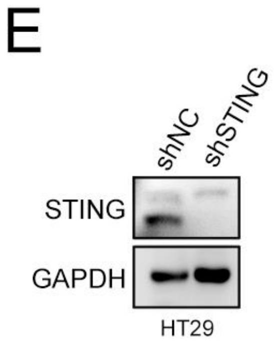
E
shNC
shSTING
STING
GAPDH
HT29

Fig. 2E

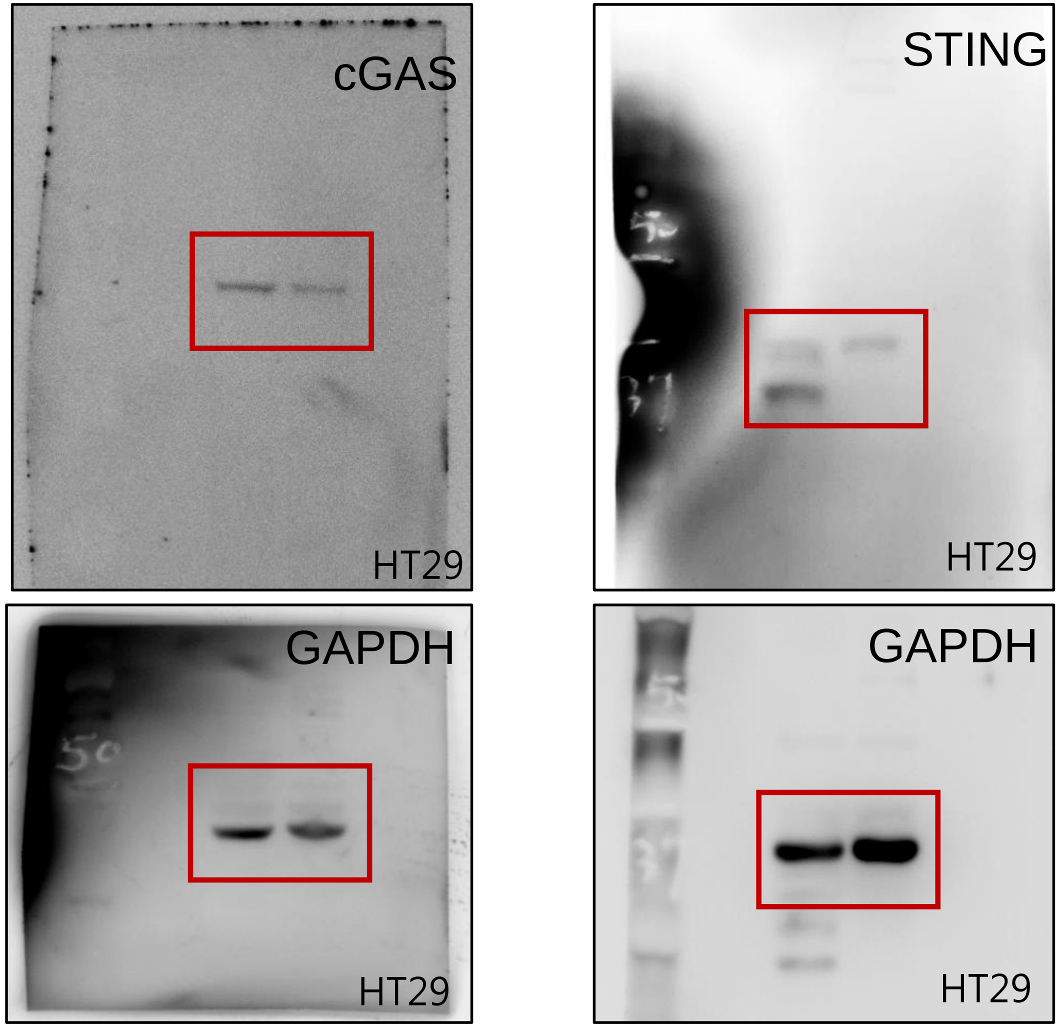
cGAS
HT29
STING
HT29
GAPDH
HT29
GAPDH
HT29

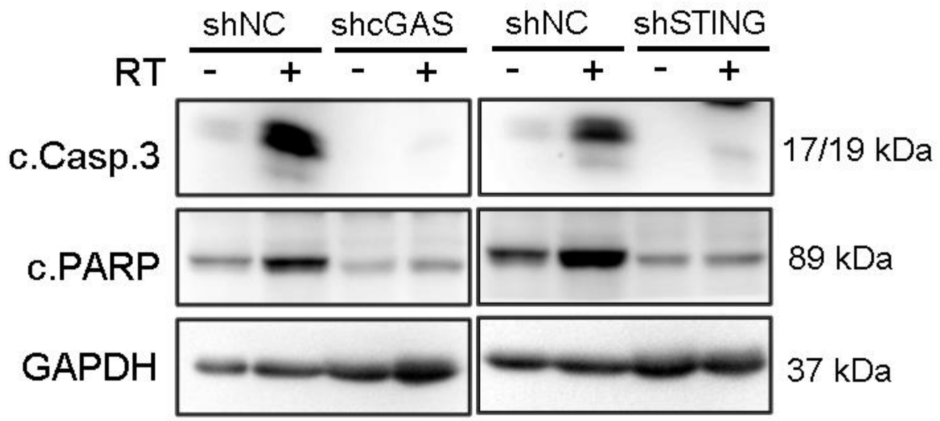

shNC
shcGAS
shNC
shSTING
RT
- + - + - + - +
c.Casp.3
c.PARP
GAPDH
17/19 kDa
89 kDa
37 kDa

Fig. 2F

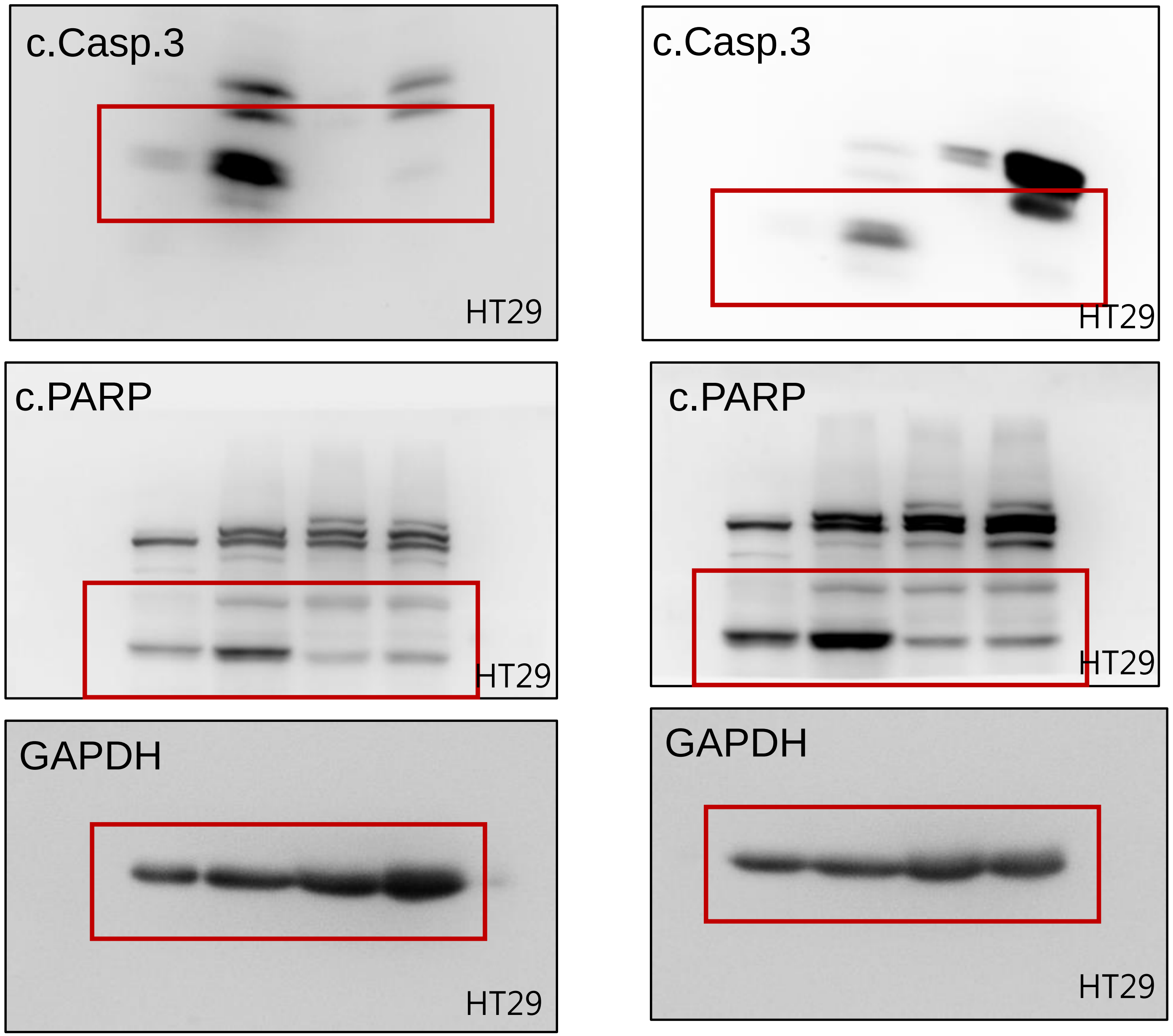

c.Casp.3
HT29
c.Casp.3
HT29
c.PARP
HT29
c.PARP
HT29
GAPDH
HT29
GAPDH
HT29

Fig. 2G

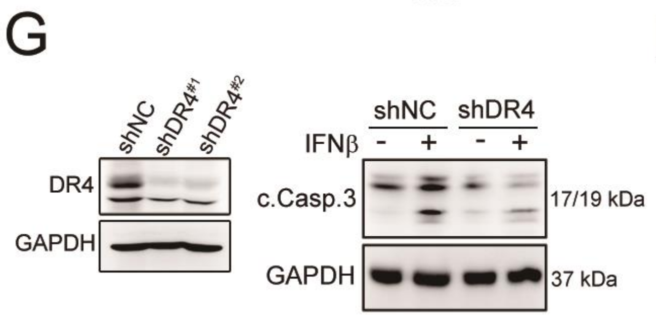
G
shNC
shDR4#1
shDR4#2
DR4
GAPDH
shNC
shDR4
IFNβ
-
+
-
+
c.Casp.3
17/19 kDa
GAPDH
37 kDa

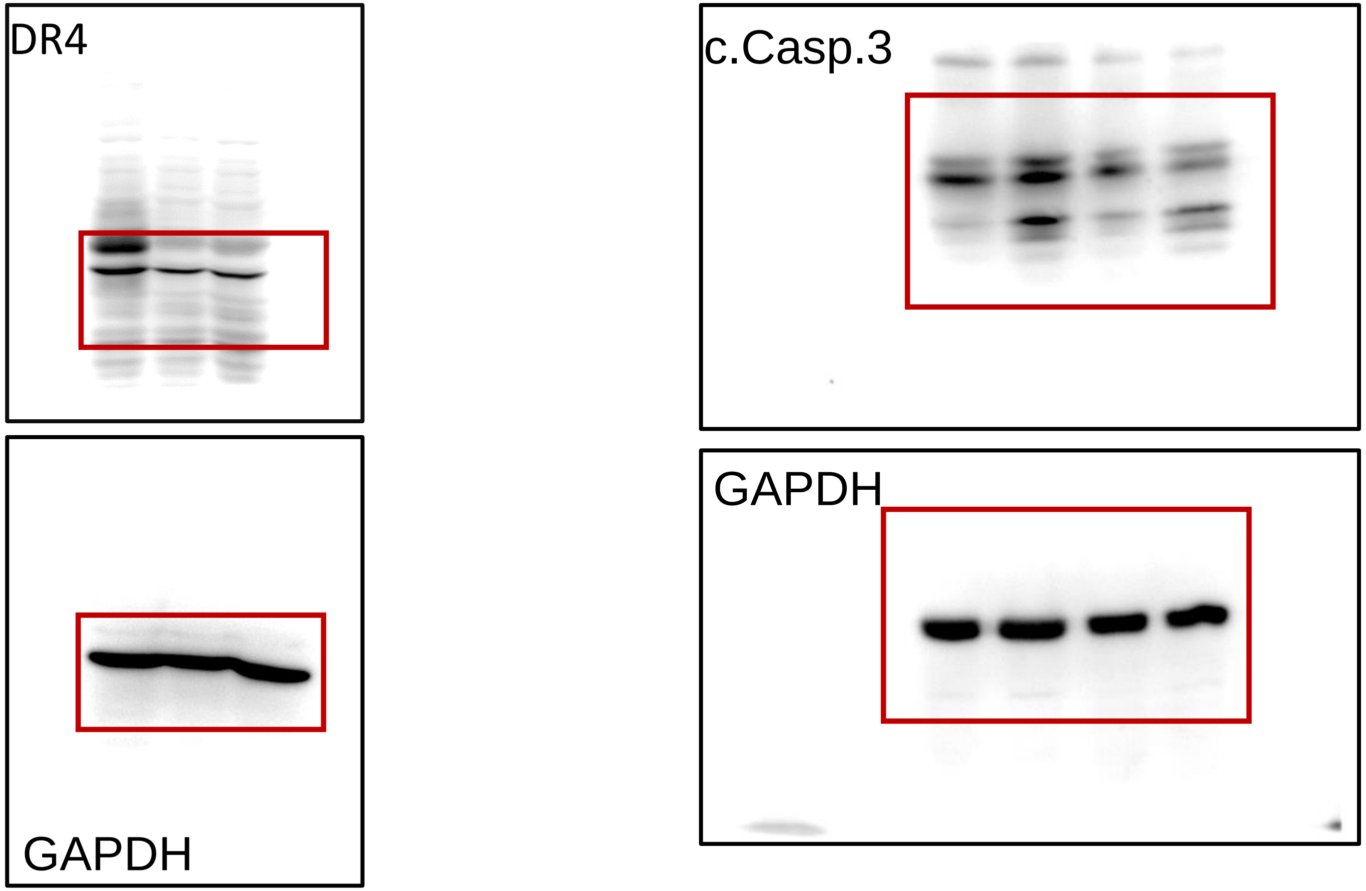
DR4
GAPDH
c.Casp.3
GAPDH

Fig. 2H

G

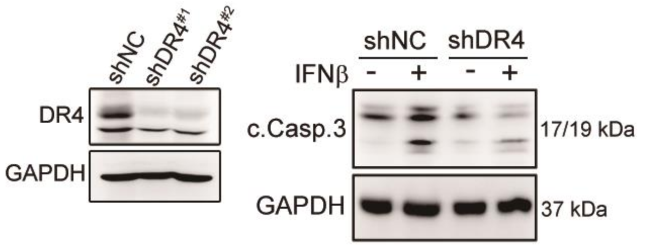

shNC
shDR4#1
shDR4#2
DR4
GAPDH
shNC
shDR4
IFNβ
- + - +
c.Casp.3
17/19 kDa
GAPDH
37 kDa

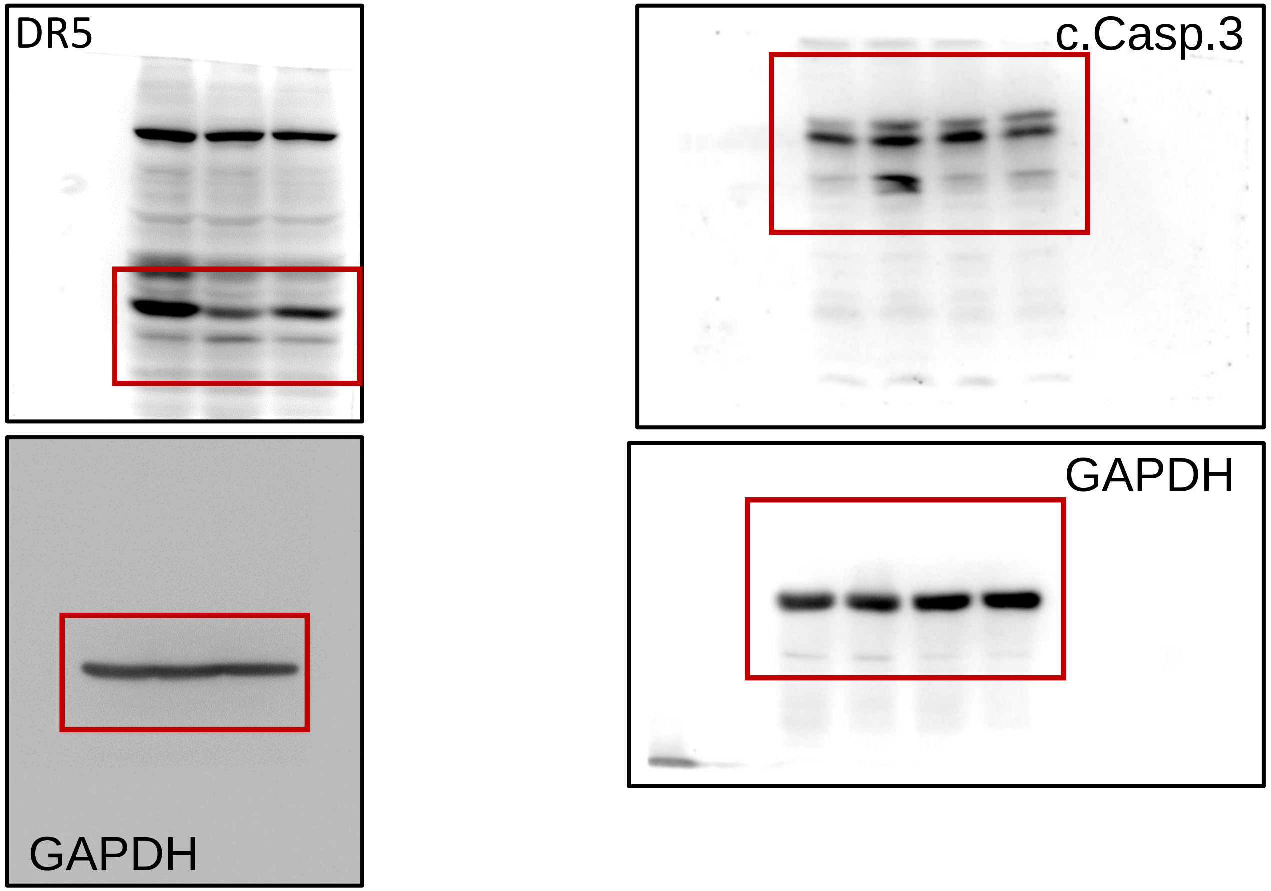

DR5
GAPDH
c.Casp.3
GAPDH

Fig. 2I

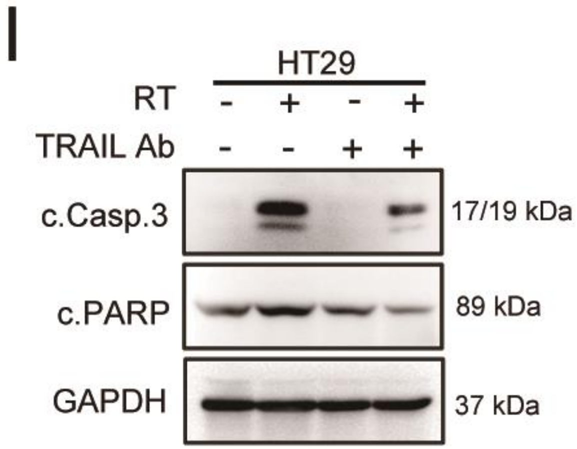
I
HT29
RT - + - +
TRAIL Ab - - + +
c.Casp.3
17/19 kDa
c.PARP
89 kDa
GAPDH
37 kDa

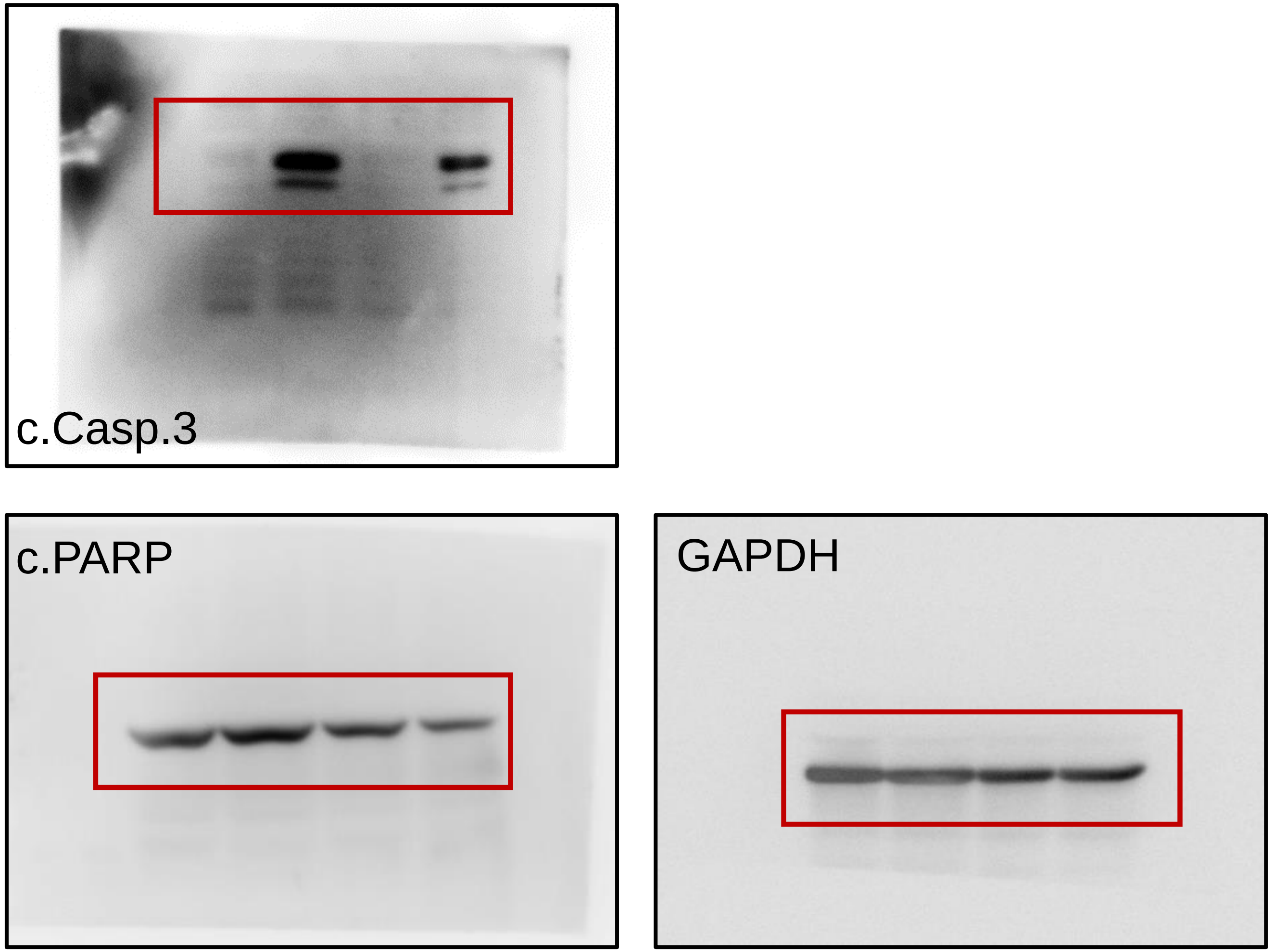
c.Casp.3
c.PARP
GAPDH

C

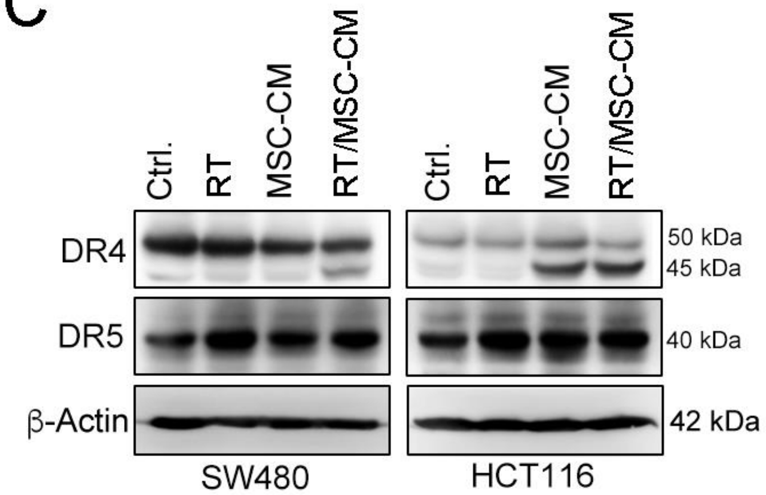

Ctrl.
RT
MSC-CM
RT/MSC-CM
DR4
DR5
β-Actin
50 kDa
45 kDa
40 kDa
42 kDa
SW480
HCT116

Fig. 3C

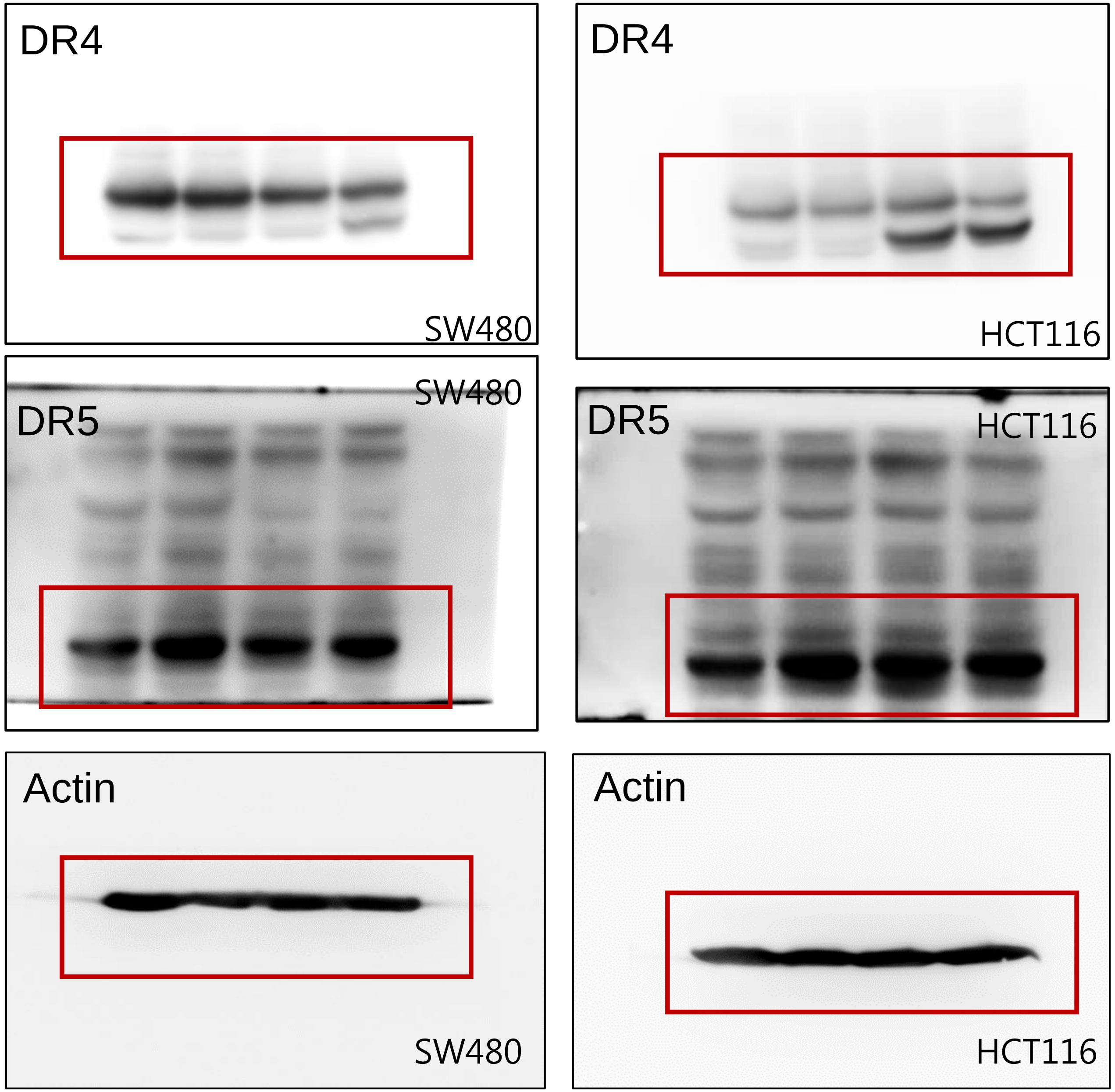

DR4
SW480
DR4
HCT116
DR5
SW480
DR5
HCT116
Actin
SW480
Actin
HCT116

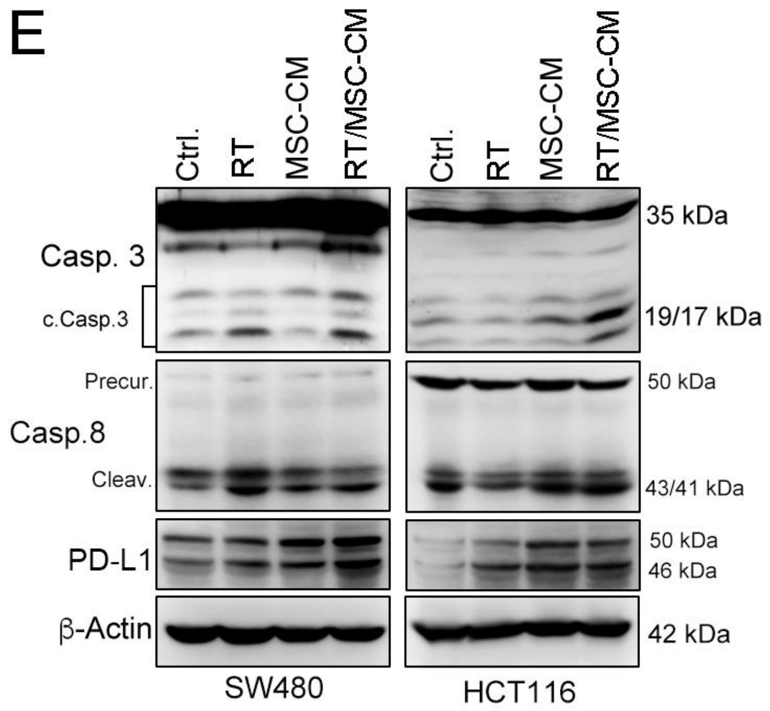
E
Ctrl.
RT
MSC-CM
RT/MSC-CM
Ctrl.
RT
MSC-CM
RT/MSC-CM
Casp. 3
c.Casp.3
35 kDa
19/17 kDa
Precur.
Casp.8
Cleav.
50 kDa
43/41 kDa
PD-L1
50 kDa
46 kDa
β-Actin
42 kDa
SW480
HCT116

Fig. 3E

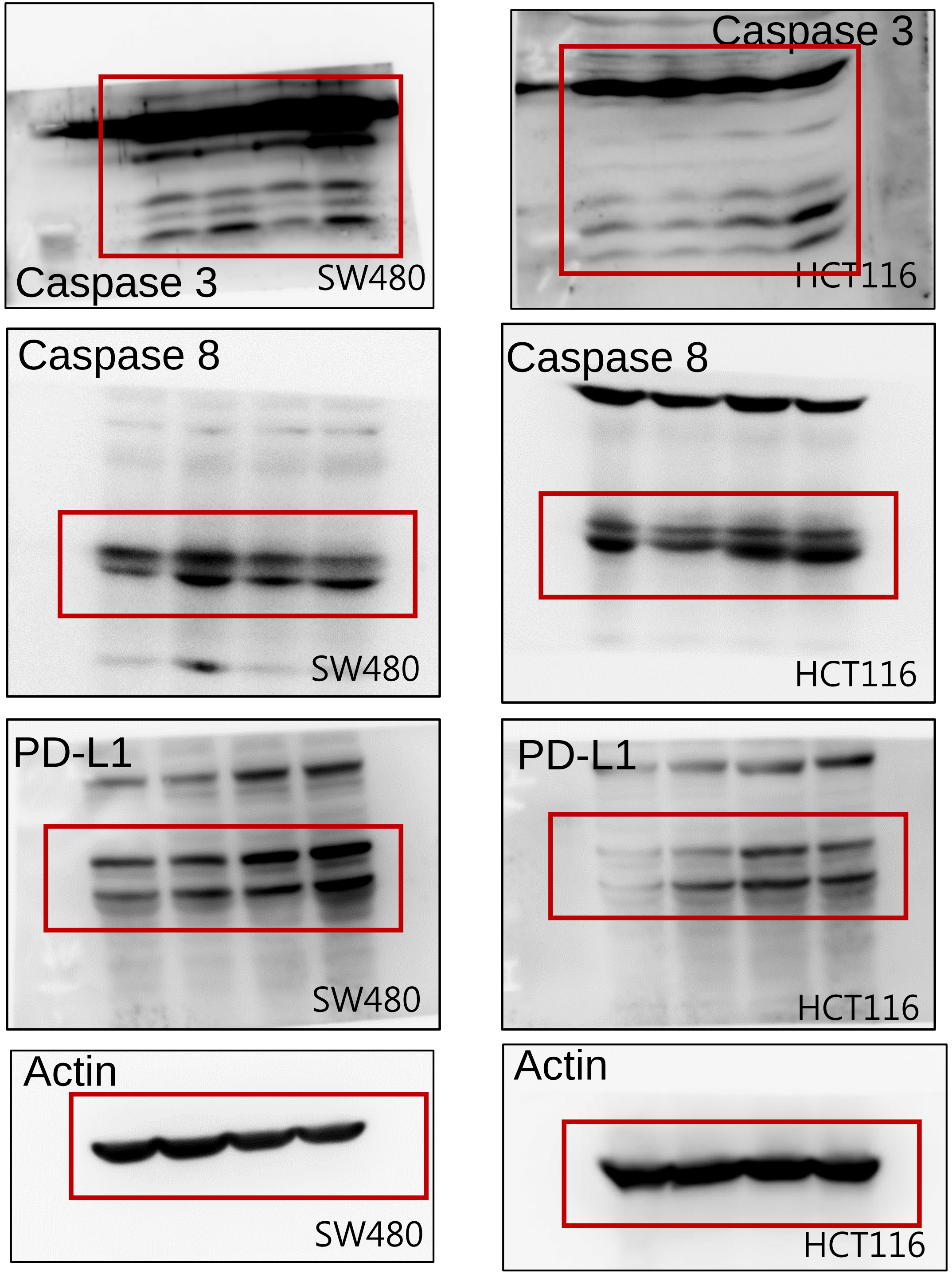
Caspase 3
SW480
Caspase 3
HCT116
Caspase 8
SW480
Caspase 8
HCT116
PD-L1
SW480
PD-L1
HCT116
Actin
SW480
Actin
HCT116

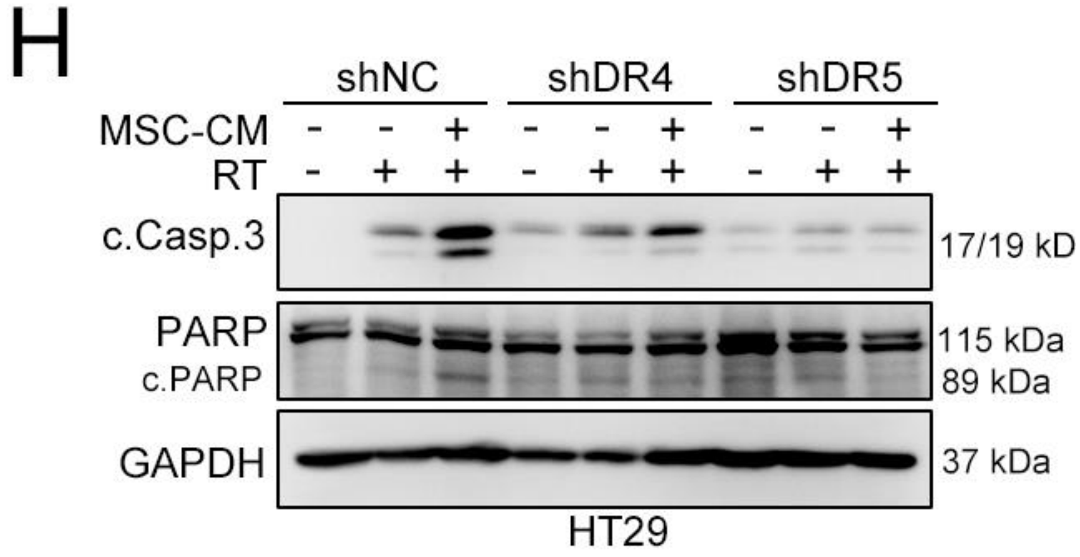
H
shNC
shDR4
shDR5
MSC-CM - - + - - + - - +
RT - + + - + + - + +
c.Casp.3
17/19 kD
PARP
c.PARP
115 kDa
89 kDa
GAPDH
37 kDa
HT29

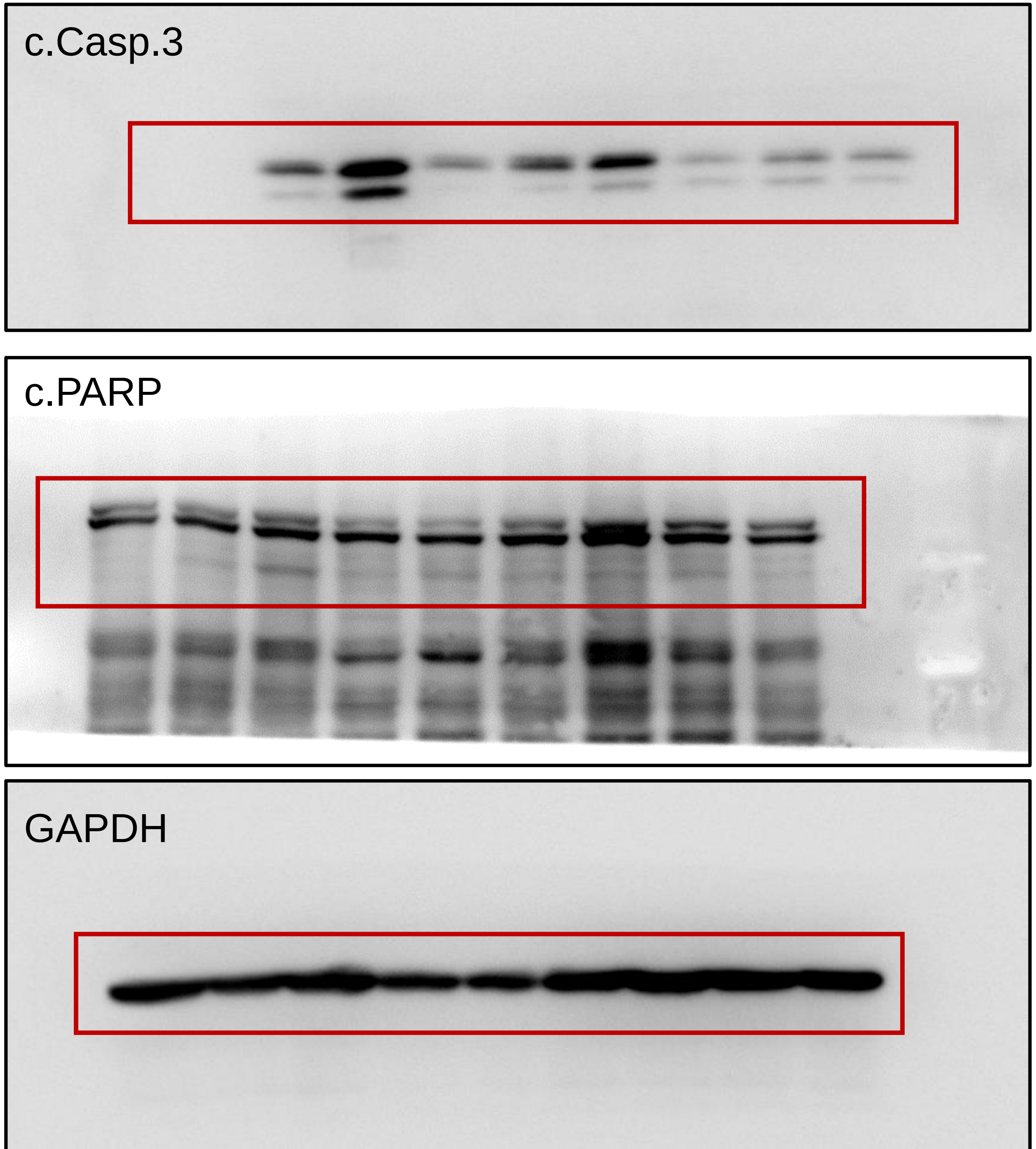
c.Casp.3
c.PARP
GAPDH

Fig. 3H

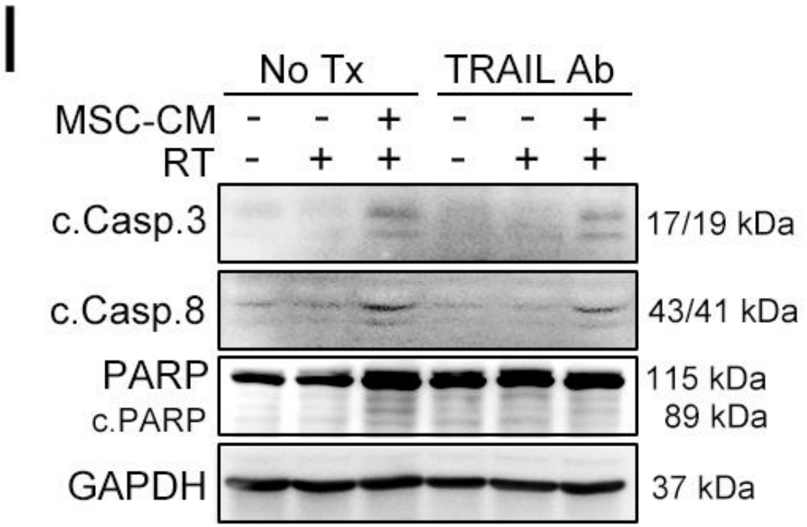
I
No Tx
TRAIL Ab
MSC-CM - - + - - +
RT - + + - + +
c.Casp.3
17/19 kDa
c.Casp.8
43/41 kDa
PARP
c.PARP
115 kDa
89 kDa
GAPDH
37 kDa

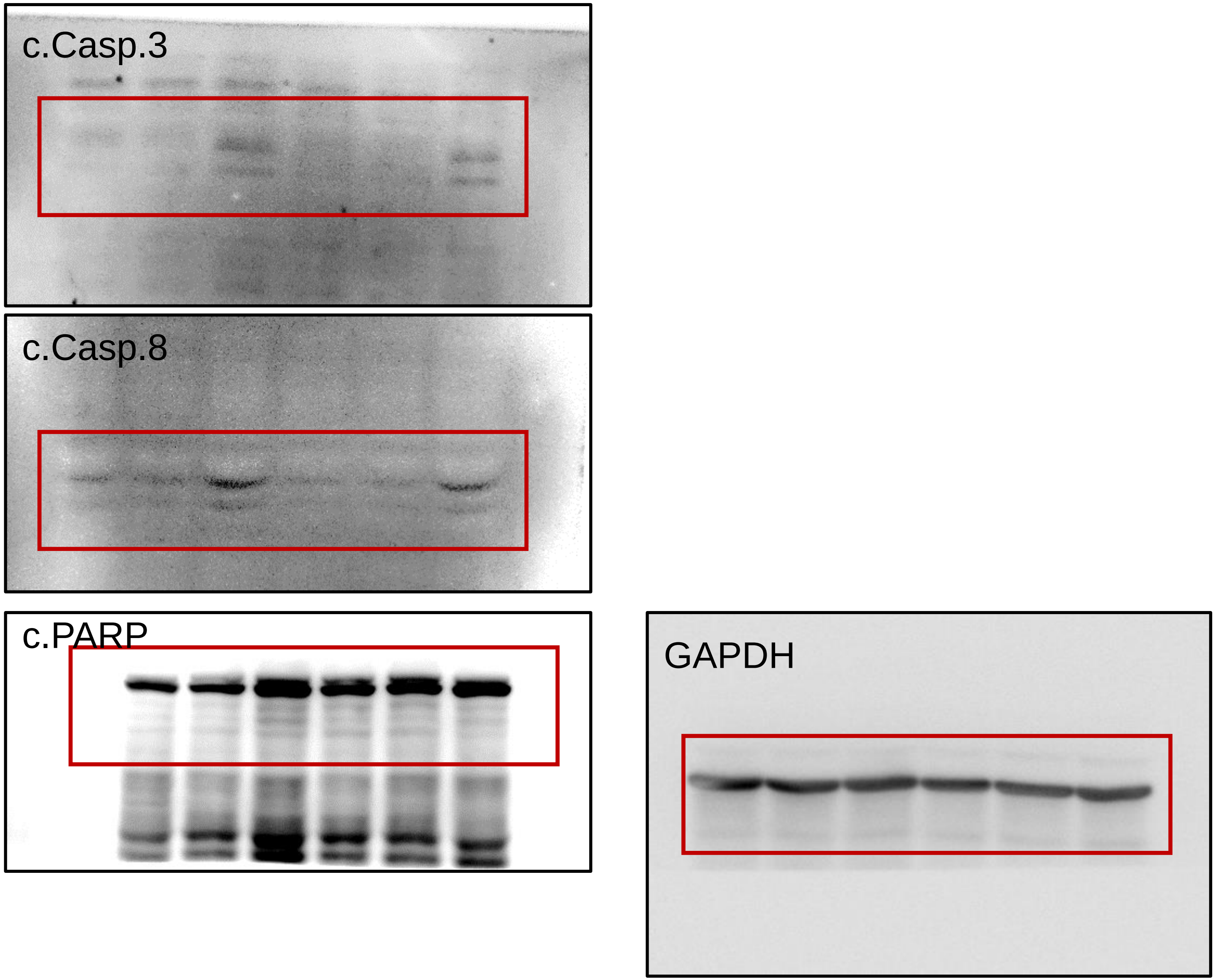
c.Casp.3
c.Casp.8
c.PARP
GAPDH

Fig. 3I

E

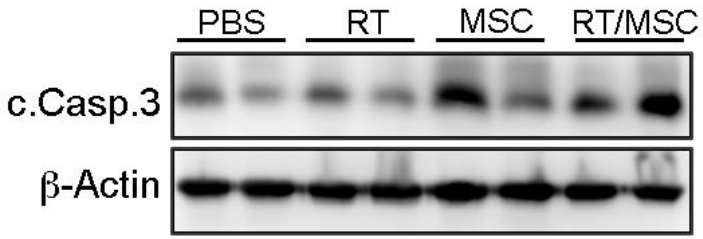

Fig. 4E

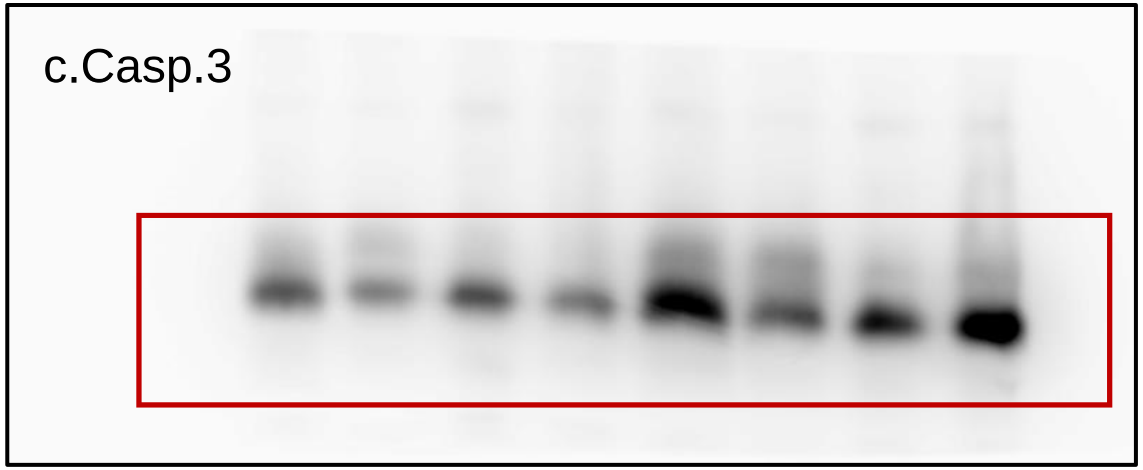

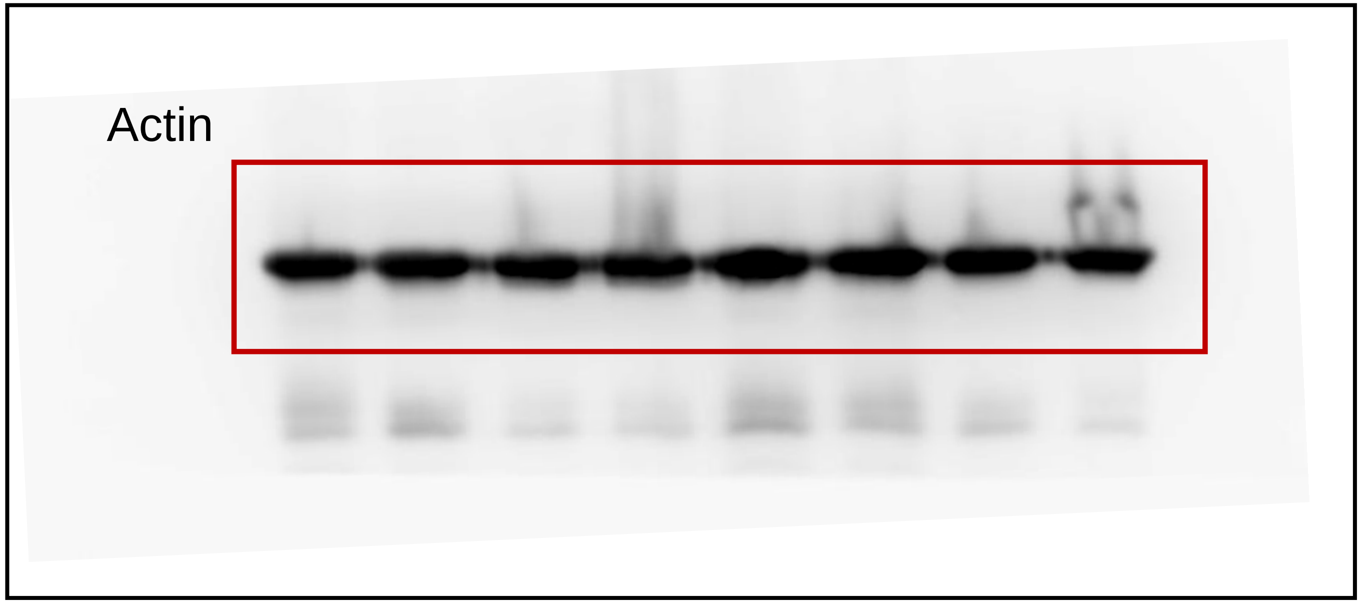

I

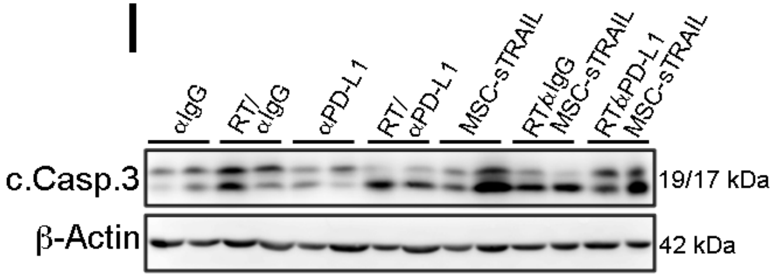
αIgG
RT/ αIgG
αPD-L1
RT/ αPD-L1
MSC-sTRAIL
RT&αIgG MSC-sTRAIL
RT&αPD-L1 MSC-sTRAIL
c.Casp.3
β-Actin
19/17 kDa
42 kDa

Fig. 5E

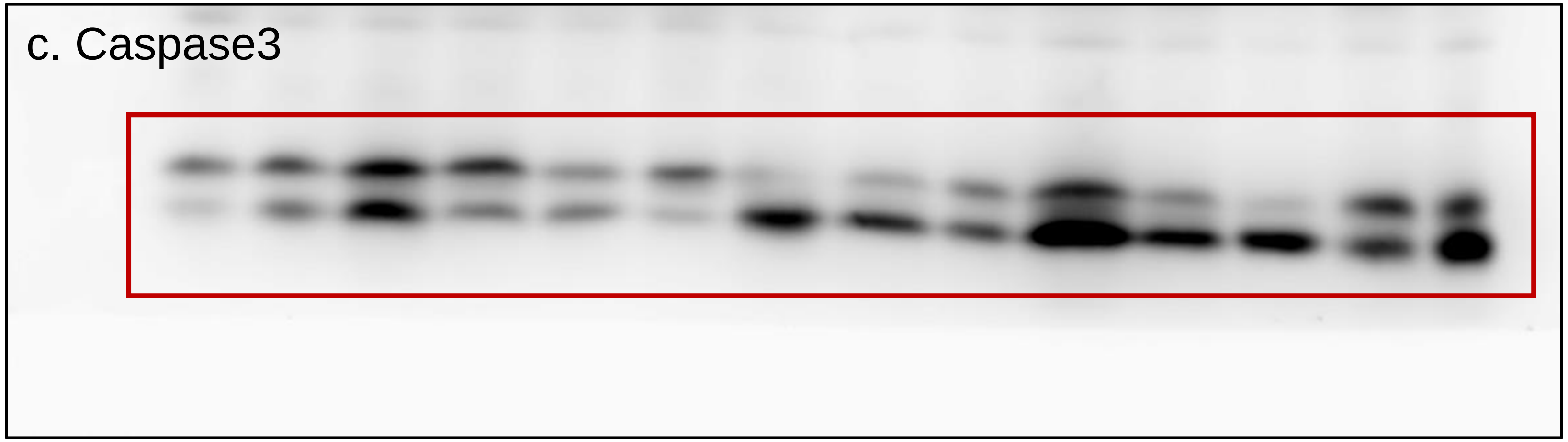
c. Caspase3

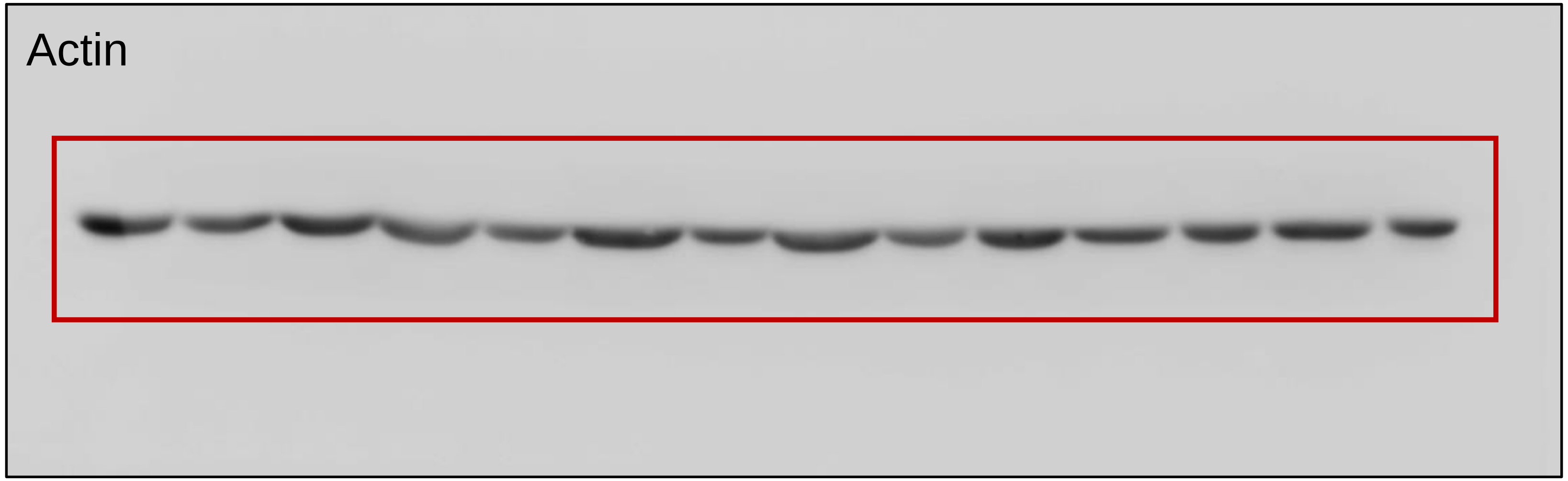
Actin

Fig. S1C

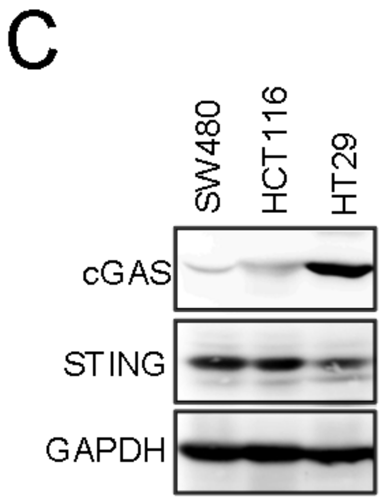

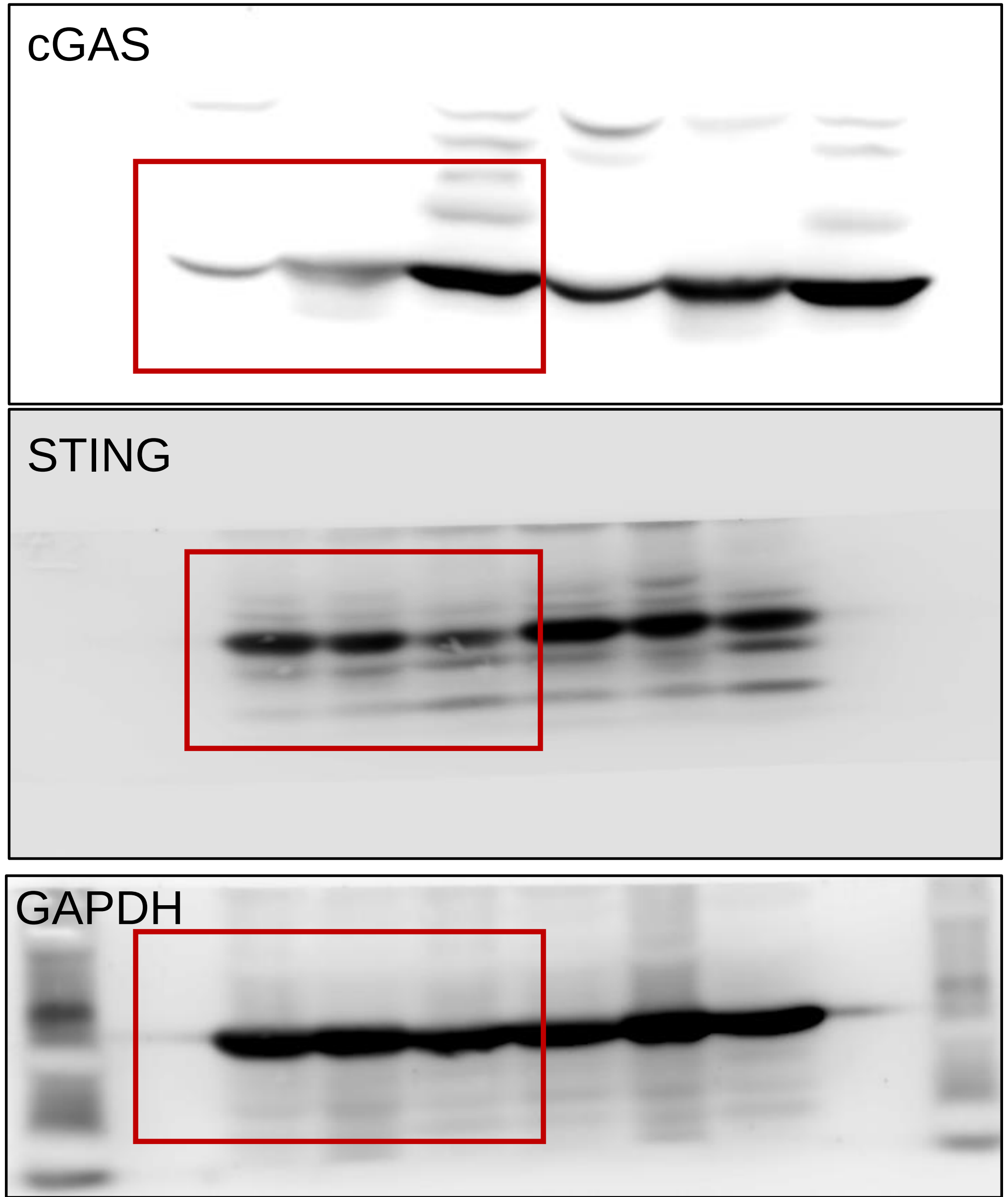

Fig. S2B

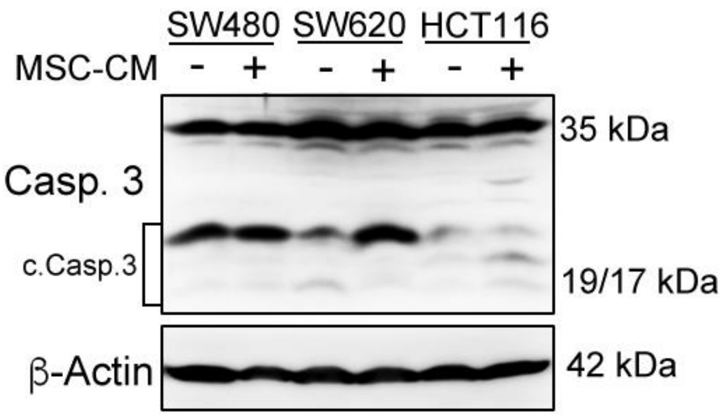
SW480 SW620 HCT116
MSC-CM - + - + - +
Casp. 3
c.Casp.3
β-Actin
35 kDa
19/17 kDa
42 kDa

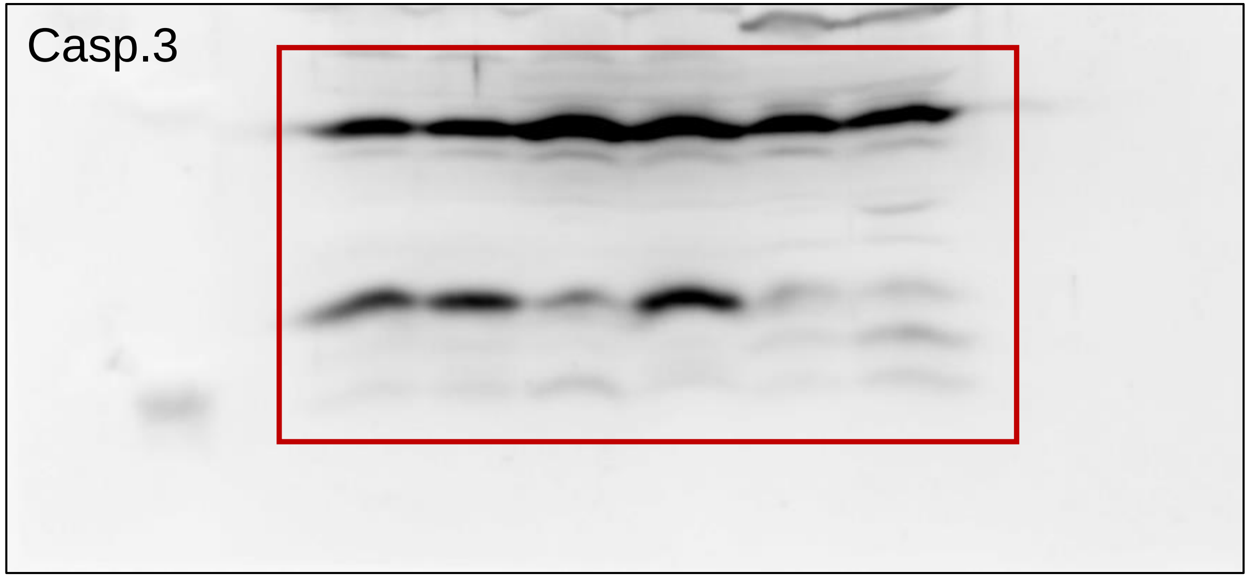
Casp.3

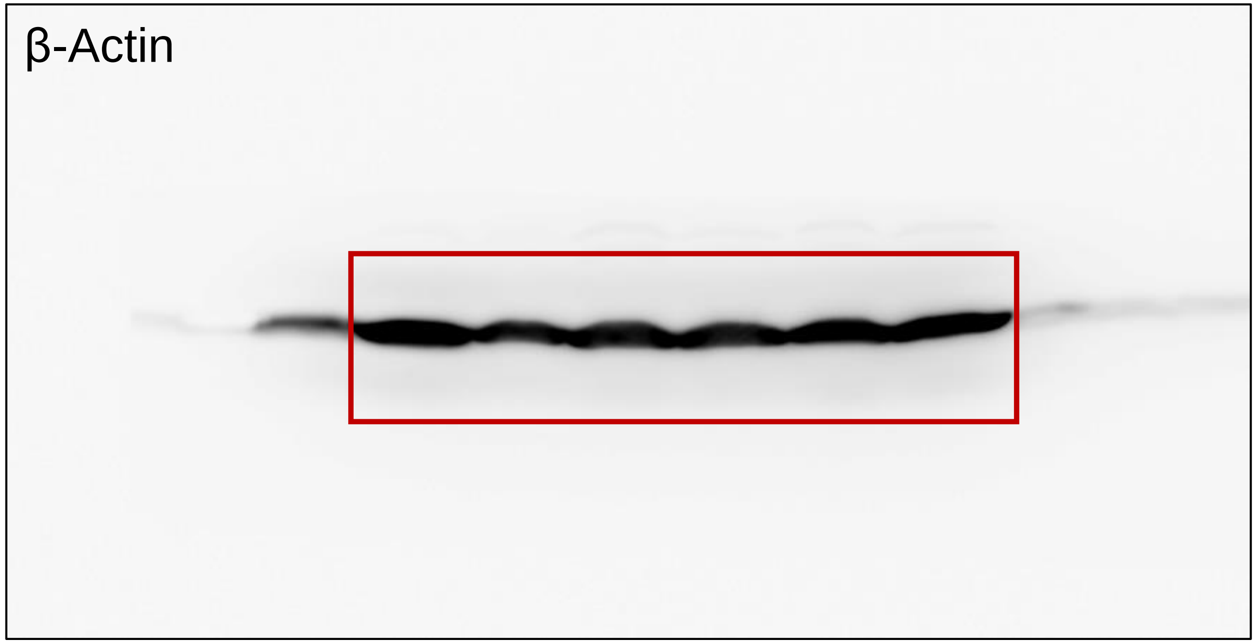
β-Actin
